# Cold atmospheric plasma potentiates ferroptosis via EGFR(Y1068)-mediated dual axes on GPX4 among triple negative breast cancer cells

**DOI:** 10.7150/ijbs.105455

**Published:** 2025-01-01

**Authors:** Xiaofeng Dai, Ziyao Xu, Xinyu Lv, Chao Li, Ruichen Jiang, Danjun Wang, Ming Xi, Tian Li

**Affiliations:** 1 National Local Joint Engineering Research Center for Precision Surgery & Regenerative Medicine, Shaanxi Provincial Center for Regenerative Medicine and Surgical Engineering, The First Affiliated Hospital of Xi'an Jiaotong University, Xi'an Jiaotong University, Xi'an 710061, China.; 2 Senior Department of General Surgery, the First Medical Center of Chinese PLA General Hospital, Beijing, China.; 3 Wuxi School of Medicine, Jiangnan University, Wuxi 214122, China.; 4 Tangshan Vocational & Technical College, Tangshan, China.; 5 Department of Oncology, Affiliated Hospital of Inner Mongolia Medical University, Huhehot 010050, China.; 6 Beijing University of Chinese Medicine, Beijing 100029, China.; 7 Department of Orthopaedics, China-Japan Friendship Hospital, Beijing 100029, China.; 8 Tianjin Key Laboratory of Acute Abdomen Disease-Associated Organ Injury and ITCWM Repair, Institute of Integrative Medicine of Acute Abdominal Diseases, Tianjin Nankai Hospital, Tianjin Medical University, 8 Changjiang Avenue, Tianjin 300100, China.

**Keywords:** cold atmospheric plasma, ferroptosis, triple negative breast cancer, EGFR(Y1068), sorafenib

## Abstract

Cold atmospheric plasma (CAP) has been proposed as an emerging onco-therapeutics that can specifically kill cancer cells without harming healthy cells. Here we explore its potency in triggering ferroptosis in transformed cells using triple negative breast cancer as the disease model. Through the whole transcriptome sequencing, mass spectrometry analysis, point mutation, and a series of* in vitro* and* in vivo* molecular assays, we identified two signaling axes centered at EGFR(Y1068), i.e., EGFR-TRIM25-KEAP1/SIAH2-NRF2 and EGFR-p38-NRF2, which suppressed GPX4 at both transcriptional and translational levels. We, in addition, demonstrated the potency of CAP in synergizing with Sorafenib towards enhanced selectivity against cancer cells via initiating ferroptosis. We are the first to systematically clarify the molecular mechanism of GPX4-dependent ferroptosis induced by CAP, and propose the feasibility of activating EGFR instead of suppressing it as well as the benefits of resolving tumors by coupling CAP with ferroptosis-inducing agents. The identified signaling axis is applicable to all cancers harboring EGFR that deserve intensive investigations.

## Background

Despite the declining rate of cancer mortality over the past two decades, breast cancer incidence has been kept increasing with 300590 new cases and 43700 death events estimated to occur in the United States in 2023^1^. Breast carcinoma is highly heterogeneous, with triple negative breast cancer (TNBC) being the most aggressive that does not respond well to canonical hormonal or targeted therapeutic strategies due to the lack of surface receptors such as estrogen receptor (ER), progesterone receptor (PR) and human epithelial receptor 2 (HER2)^2^, ^3^. Despite consecutive reports on agents targeting TNBCs^4^, standard-of-care for TNBCs still relies on chemotherapies due to the limited responsive spectrum of these therapeutics and their unavoidable side effects. This makes investigations into novel onco-therapeutic modalities capable of specifically resolving TNBC tumors with little adverse consequences highly imperative.

Cold atmospheric plasma (CAP) belongs to the fourth state of matter and is a cocktail of reactive oxygen and nitrogen species (RONS) such as hydroxyl radical (OH•), hydrogen peroxide (H_2_O_2_), ozone (O_3_), singleton oxygen (O), superoxide (O^2-^), nitric oxide (NO), and nitrite in the form of anion or proton (OONO^-^, ONOOH)^5^, ^6^. CAP has showcased its efficacy in selectively resolving various malignant tumors including, e.g., melanomas^7^, ^8^, pancreatic cancers^9^, liver carcinomas^10^, prostate cancers^11^, bladder cancers^12^, and breast tumors 6, 13, 14 since its initial discovery on the specific induction of cancer cell death in 2007. In the clinics, the use of CAP secured the life of a 75-year-old advanced pancreatic cancer patient in 2016, and saved the life of a 33-year-old relapsed peritoneal sarcoma patient in 2019^15^. These successful case studies have led to the initiation of the first clinical trial exploring the potency of CAP as an onco-therapy in 2019 (NCT04267575). This phase I trial recruited 20 advanced solid tumor patients of various types, and lasted for 2 years. By the end of this study, 17 patients who had been diagnosed with 2-3 month life span in the initial phase of this trial were still alive^16^, ^17^, suggesting the feasibility of applying CAP for cancer treatment.

Incremental evidence has suggested the advantages of CAP in treating cancers over many existing modalities for cancer treatment. The most attractive feature is the specificity of CAP in killing transformed cells without harming their healthy peers. That means, in contrast to chemotherapy that kills fast growing cells without differentiating healthy and malignant cells, CAP conveys little toxicity; compared with radiotherapy that relies on physical targeting to ablate tumor cells, CAP by itself can recognize cancer cells; compared with targeted therapy that has fixed molecular targets, CAP is highly plastic that does not rely on any single target or molecular mechanism to deliver desirable therapeutic outcome on appropriate dosage^18^; and compared with immune therapy, CAP can sensitize the micro-environment of solid tumors for enhanced vulnerability of cancer cells to immune therapy^19^. Given these multifaceted advantages, increasing efforts have been devoted to decipher the mechanisms driving these unique characteristics of CAP in ablating cancers.

This study focuses on the cell death programs. Besides apoptosis^6^, ^8^, ^12^-^14^, autophagy^20^ and immunogenic cell death (ICD)^21^-^24^ have also been implicated in CAP-triggered programmed cancer cell death. These suggest the co-existence of multiple death programs and necessitate investigations on novel death events triggered by CAP among cancer cells. The role of CAP in triggering ICD^23^, the potentiating power of lipid peroxidation to ICD^25^, and the intrinsic connection between lipid peroxidation and ferroptosis motivated us to focus on ferroptosis. Ferroptosis is a novel form of regulated cell death that occurs via increased lipid peroxidation and iron accumulation. Compared with normal cells, cancer cells have differential expression and activities of several iron-related proteins that participate in many critical events responsible for carcinogenesis such as DNA repair and epigenetic remodeling^26^. Such a distinct proteomic profile drives a higher intracellular iron level in cancer cells to actionize these iron-dependent proteins towards accelerated tumorigenesis. Thus, strategies relying on iron depletion or targeting iron metabolism such as iron chelators and iron oxide nanoparticles have been proposed with potent anti-tumor efficacy, some of which have already been under the clinical evaluation^27^. On the other hand, ferroptosis is featured by reduced size of mitochondria and a necrotic morphology as a result of ROS accumulation^28^. Given the intrinsic redox-modulatory role of CAP and elevated level of irons in malignant cells, it is natural to assume that CAP can induce the ferroptosis of cancer cells and may represent a promising recipe for TNBC management.

Motivated as such, we explored the efficacy and selectivity of CAP in potentiating ferroptosis among TNBC cells, and identified two molecular axes centered at activating the tyrosine 1068 site (Y1068) of epidermal growth factor receptor (EGFR). Specifically, activated EGFR(Y1068) in response to CAP induced the ubiquitination of nuclear factor erythroid 2-related factor 2 (NRF2), a master molecule controlling the cellular anti-oxidant defense mechanism via tripartite motif containing 25 (TRIM25)/kelch like ECH associated protein 1 (KEAP1) and p38 signaling; and reduced level of NRF2 led to suppressed glutathione peroxidase 4 (GPX4) at both transcriptional and translational levels, resulting in ferroptosis.

## Results

### CAP triggers ferroptosis among TNBC cells

**Figure 1:**
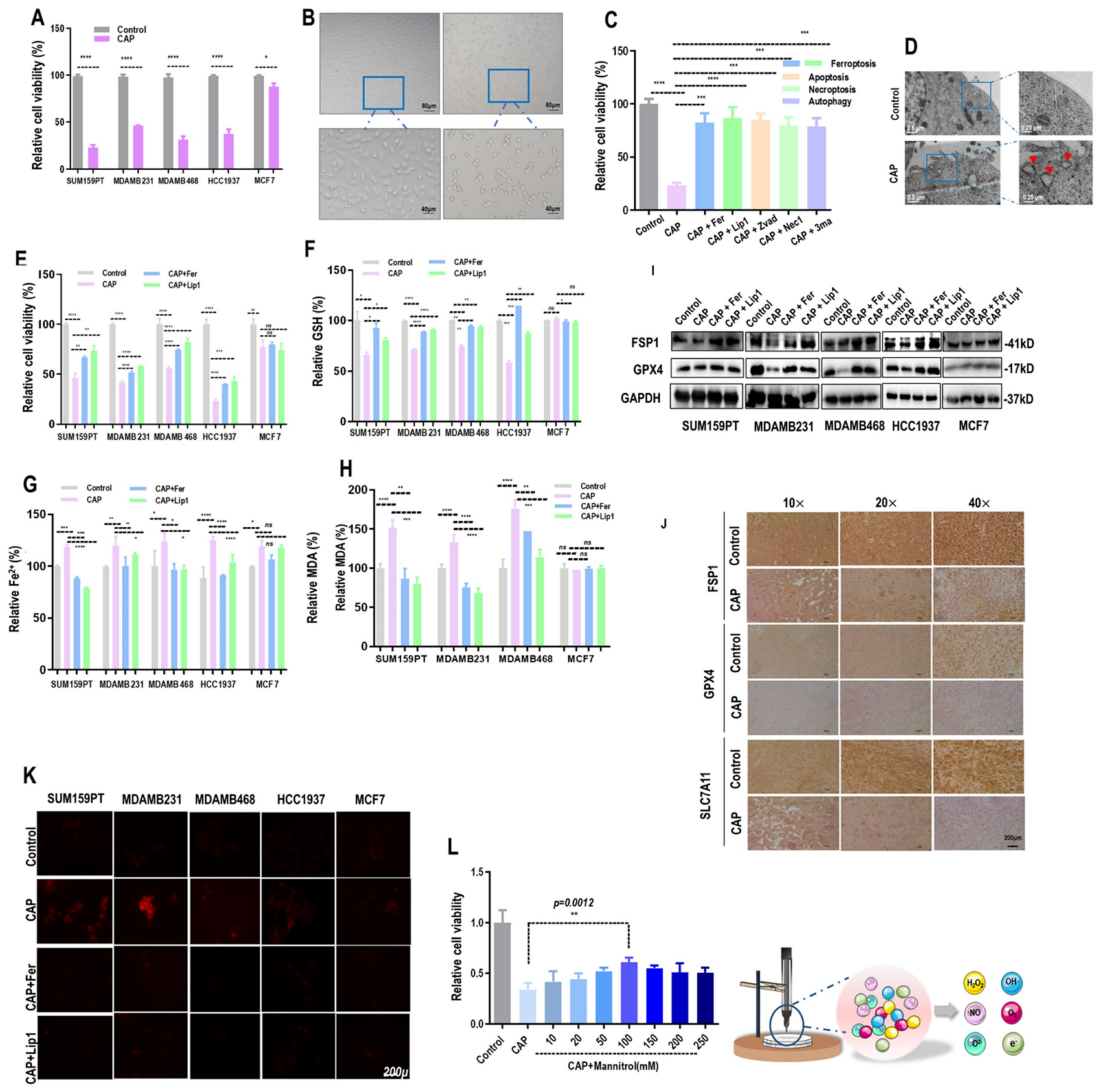
** CAP triggers ferroptosis among TNBC cells. (A)** Viabilities of breast cancer cells in response to CAP treatment.** (B)** Phenotypic images of SUM159PT cells in response to CAP treatment under microscopy.** (C)** Viabilities of breast cancer cells in response to CAP treatment and different death inhibitors. Fer (ferrostatin-1) and Lip1 (liproxstatin-1) are inhibitors of ferroptosis, Zvad (Z-VAD-FMK), Nec1 (necrostatin-1), 3ma (3-methyladenine) are inhibitors of apoptosis, necroptosis and autophagy, respectively.** (D)** Subcellular structure images of SUM159PT cells in response to CAP treatment under electron microscopy.** (E)** Relative cell viabilities,** (F)** relative reductive glutathione (GSH) percentage, **(G)** relative Fe^2+^ percentage, and **(H)** relative malondialdehyde (MDA) percentage in different breast cancer cells in response to CAP and ferroptosis inhibitors. **(I)** Western blot showing the expression of ferroptosis markers GPX4, FSP1 in response to CAP and ferroptosis inhibitors in different breast cancer cell lines. **(J)** Immunohistochemistry blotting showing the expression of ferroptosis markers GPX4, FSP1, SLC7A11 in response to CAP in mice inoculated with SUM159PT cells. **(K)** ROS quantification assay showing cellular redox status in response to CAP and ferroptosis inhibitors in different breast cancer cell lines.** (L)** Illustrative diagram of primary CAP components, and viabilities of cells in response to CAP treatment without and with OH• being quenched by mannitrol at different concentrations. SUM159PT, MDAMB231, MDAMB468, HCC1937 are TNBC cells, MCF7 is a luminal cell line. * represents statistical significance. Significance level: * <0.1, ** <0.01, ***<0.001, ****<0.0001.

CAP selectively reduced 60-90% viability of TNBC cells (SUM159PT, MDAMB231, MDAMB468, HCC1937) with statistical significance (*p*<0.0001), whereas that of MCF7 was reduced approximately 10% (**Figure 1A**). The morphology of SUM159PT cells became blurry with reduced transparency after CAP treatment (**Figure 1B**). By supplementing cells with inhibitors of varied cell death programs including ferrostatin-1 (Fer) and liproxstatin-1 (Lip1) for ferroptosis, Z-VAD-FMK (Zvad) for apoptosis, necrostatin-1 (Nec1) for necroptosis, and 3-methyladenine (3ma) for autophagy, separately, we observed significantly restored viability of SUM159PT cells. Among these inhibitors, Lip1 or Fer showed the most evident restoration (**Figure 1C**), implicating that ferroptosis may be a leading cause of CAP-induced cancer cell death. Under electron microscopy, we observed shrunk mitochondria with condensed membrane densities, reduced crista and diminished outer membrane rupture (**Figure 1D**).

The triggering of ferroptosis by CAP was specific to TNBC cells, as the relative viability (**Figure 1E, Supplementary Figure 2A, 2B**), GSH percentage (**Figure 1F**), Fe^2+^ percentage (**Figure 1G**), and malondialdehyde (MDA) percentage (**Figure 1H**) were significantly altered in response to CAP treatment and restored by ferroptosis inhibitors in TNBC cells (SUM159PT, MDAMB231, MDAMB468, HCC1937). However, these variables stayed invariant after CAP treatment or treated with ferroptosis inhibitors in non-TNBC cells (MCF7). On the other hand, supplementing cells with ferroptosis activators brequinar (BQR) or RAS-selective lethal 3 (RSL3) created synergies with CAP in inducing TNBC cell ferroptosis (**Supplementary Figure 2C**, **2D**). In addition, the levels of proteins characterizing ferroptosis, i.e., GPX4 and ferroptosis suppressor protein 1 (FSP1), were suppressed by CAP (**Supplementary Figure 2E**, **2F**) and restored by Fer or Lip1 in TNBC cells (**Figure 1I**). The suppressive role of CAP on GPX4, FSP1 and SLC7A11 were confirmed using mice tumor samples (**Figure 1J**).

In consistent with the inductive role of CAP on ferroptosis, CAP elevated the redox level of TNBC cells that were quenched by ferroptosis inhibitors (**Figure 1K**). As a prominent component of CAP, OH• has also been considered as a prerequisite for generating lipid peroxyl radical (PL-OO•)^29^. By quenching OH• using mannitrol at different concentrations, we observed rewired sensitivities of SUM159PT cells to CAP-triggered death in a dose-dependent manner, suggesting the existence of OH•-induced lipid ROS accumulation and oxidation^30^, ^31^ that, consequently, initiated cell ferroptosis (**Figure 1L**).

### CAP-triggered ferroptosis occurs via activating EGFR(Y1068)

**Figure 2:**
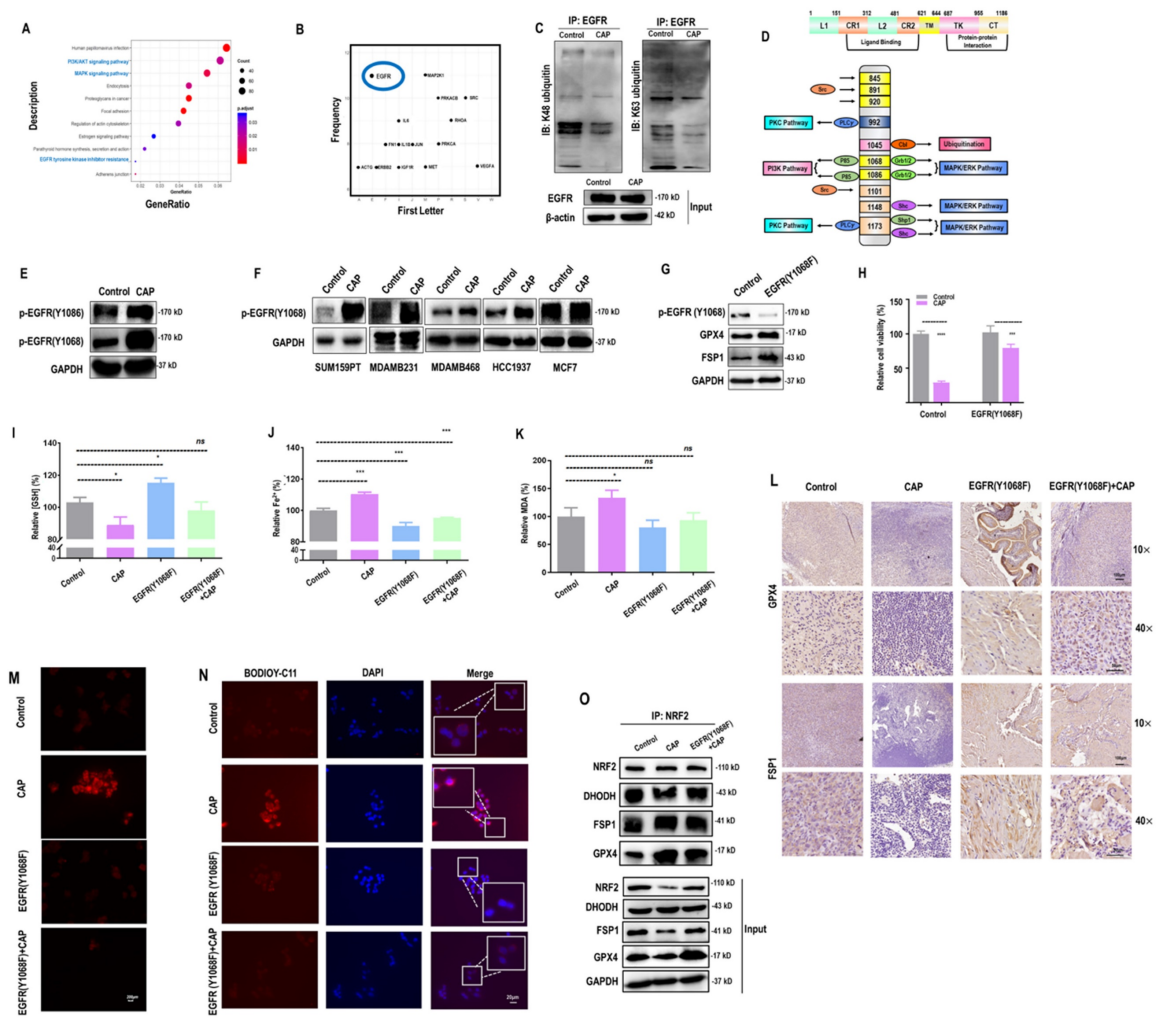
** CAP-triggered ferroptosis occurs via EGFR(Y1068) activation. (A)** KEGG pathways enriched with genes differentially expressed between samples receiving or not CAP treatment using the whole transcriptome data retrieved from^14^.** (B)** Frequency map showing proteins differentially expressed between samples with and without CAP treatment using the whole transcriptome data retrieved from^14^. **(C)** EGFR K48 and K63 ubiquitination in protein samples with and without CAP treatment.** (D)** Illustrative diagram showing downstream pathways associated with different EGFR phosphorylation sites.** (E)** Western blot showing the phosphorylation status of different EGFR phosphorylation sites in response to CAP exposure. **(F)** Western blot showing the expression of EGFR(Y1068) phosphorylation status in response to CAP treatment in different breast cancer cell lines.** (G)** Western blot showing the protein levels of ferroptosis markers in SUM159PT cells with and without EGFR(Y1068F) mutation.** (H)** Viabilities of cells with and without EGFR(Y1068F) mutation in response to CAP treatment.** (I)** Relative GSH percentage,** (J)** relative Fe^2+^ percentage,** (K)** relative MDA percentage in SUM159PT cells with and without EGFR(Y1068F) mutation in response to CAP treatment.** (L)** Immunohistochemistry blotting showing the protein expression of ferroptosis markers in samples obtained from mice inoculated with SUM159PT cells with and without EGFR(Y1068F) mutation in response to CAP treatment.** (M)** ROS quantification assay showing cellular redox status and **(N)** lipid ROS assay showing lipid ROS levels in SUM159PT cells with and without EGFR(Y1068F) mutation in response to CAP treatment.** (O)** Immunoprecipitation results showing interactions between NRF2 and ferroptosis markers GPX4, FSP1, DHODH before and after CAP treatment in SUM159PT cells with and without EGFR(Y1068F) mutation. * represents statistically significant. Significance level: * <0.1, ** <0.01, ***<0.001, ****<0.0001.

By analyzing the whole transcriptome data retrieved from^14^, we identified several canonical cancer-associated pathways that were perturbed by CAP including 'PI3K/AKT and MAPK signalings' and 'EGFR tyrosine kinase inhibitor resistance' (**Figure 2A**). Among proteins enriched in these differentially activated pathways between cancer and normal cells, EGFR was frequently presented (**Figure 2B**). In addition, EGFR K48 and K63 ubiquitination were both substantially reduced in response to CAP treatment (**Figure 2C**), suggesting enhanced EGFR stability and reduced NF
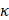
B signaling^32^ after CAP perturbation. Among the varied EGFR phosphorylation sites, Y1068 and Y1086 are associated with both PI3K and MAPK pathways (**Figure 2D**), the expression of which were both sufficiently enhanced after CAP treatment (**Figure 2E**). We selected EGFR(Y1068) for the following analysis given its relatively more distinguishable response to CAP treatment than EGFR(Y1086). Indeed, EGFR(Y1068) was over-activated after CAP treatment in TNBC cells (**Figure 2F**). Next, we generated an EGFR(Y1068F) plasmid (**Supplementary Figure 3A**) and examined its mutation efficacy (**Supplementary Figure 3B**). EGFR(Y1068) mutation enhanced the protein levels of GPX4 and FSP1 (**Figure 2G**), confirming the relevance of EGFR(Y1068) in inducing ferroptosis of SUM159PT cells. In addition, the relative viability (**Figure 2H**), GSH percentage (**Figure 2I**), Fe^2+^ percentage (**Figure 2J**) and MDA percentage (**Figure 2K**) were significantly altered after CAP treatment in SUM159PT cells, and changed in an opposite direction in SUM159PT cells that carry the EGFR(Y1068F) mutation. The opposite response of cells due to EGFR(Y1068F) mutation was restored by CAP to a level comparable with that of the wild type SUM159PT cells (**Figure 2K**). In consistent with these *in vitro* results, GPX4 and FSP1 levels were sufficiently reduced after CAP treatment, elevated in mice tumor samples carrying EGFR(Y1068F) mutation, and non-distinguishable in the 'EGFR(Y1068F)+CAP' group as compared with the control (mice inoculated with SUM159PT cells) (**Figure 2L**). The cellular redox level and lipid ROS level were sufficiently enhanced in SUM159PT cells receiving CAP treatment; but remained invariant in EGFR(Y1068F) mutants, the intensity of which was slightly reduced after CAP treatment (**Figure 2M**,** 2N**). The binding of NRF2 to GPX4 was sufficiently enhanced after CAP treatment, suggesting that NRF2 played a regulatory role on GPX4 via physical protein-protein interactions (**Figure 2O**). Enhanced interactions between NRF2 and GPX4 in EGFR(Y1068F) mutants after CAP treatment was less evident than that in the wild type SUM159PT cells (**Figure 2O**), implicating the role of EGFR(Y1068) in mediating the regulation of NRF2 on GPX4.

### EGFR(Y1068) mediates EGFR-KEAP1-NRF2-GPX4 signaling

**Figure 3:**
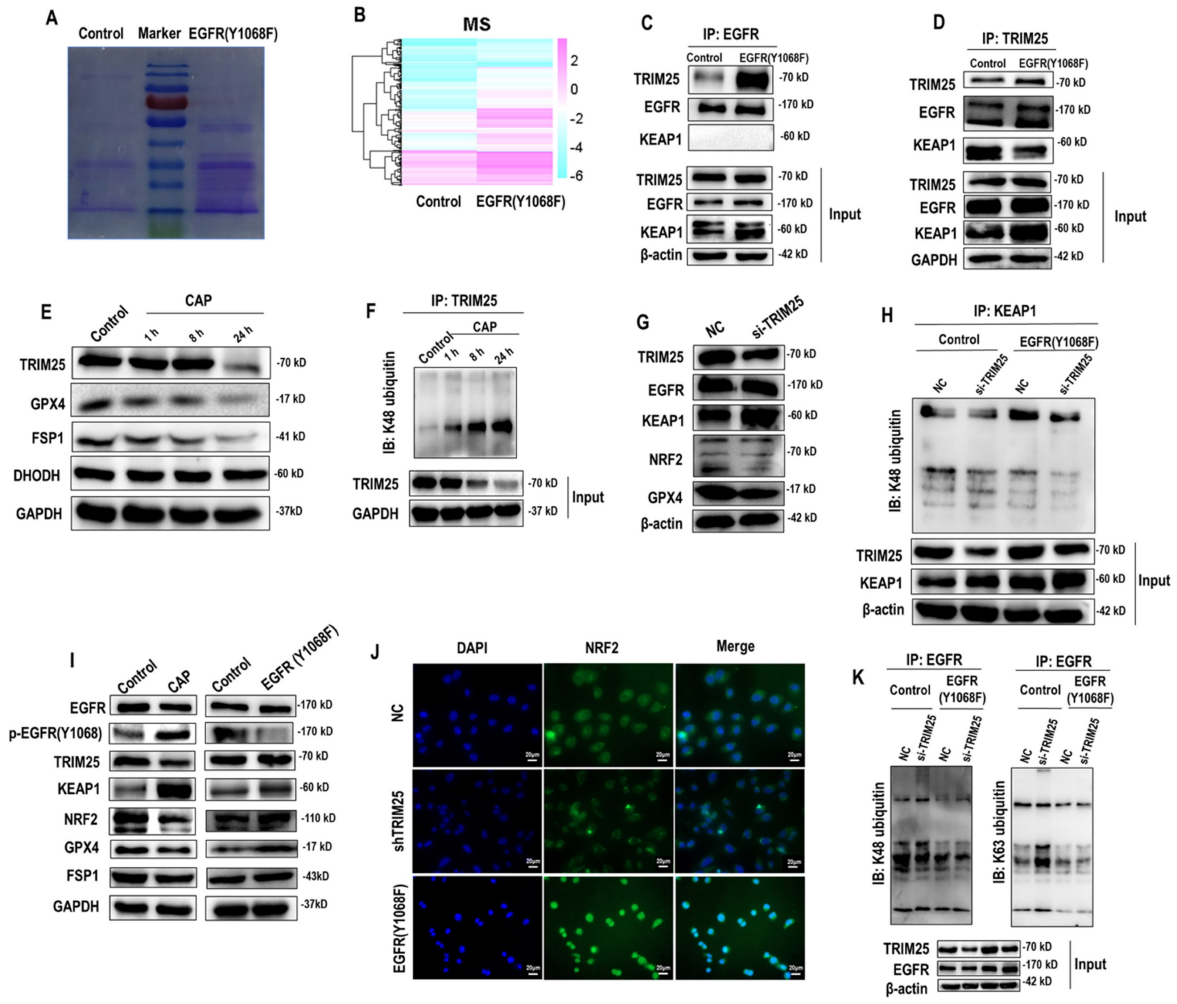
** EGFR(Y1068) mediates EGFR-KEAP1-NRF2 signaling. (A)** Agarose gel electrophoresis image showing differential protein expression profiles in SUM159PT cells with and without EGFR(Y1068F) mutation.** (B)** Heatmap showing differentially identified proteins from mass spectrometry in SUM159PT cells with and without EGFR(Y1068F) mutation.** (C)** Immunoprecipitation results showing interactions between EGFR and TRIM25, EGFR, KEAP1 in SUM159PT cells with and without EGFR(Y1068F) mutation.** (D)** Immunoprecipitation results showing interactions between TRIM25 and TRIM25, EGFR, KEAP1 in SUM159PT cells with and without EGFR(Y1068F) mutation. **(E)** Western blot showing the protein expression of TRIM25, GPX4, FSP1, DHODH in SUM159PT cells without CAP treatment or at 1 h, 8 h and 24 h post-CAP exposure.** (F)** Immunoprecipitation results showing TRIM25 K48 ubiquitination in SUM159PT cells without CAP treatment or at 1 h, 8 h and 24 h post-CAP exposure.** (G)** Western blot showing the protein expression of TRIM25, EGFR, KEAP1, NRF2, GPX4 in SUM159PT cells with and without transfection with *TRIM25* siRNAs. **(H)** Immunoprecipitation results showing KEAP1 K48 ubiquitination in SUM159PT cells with and without transfection with *TRIM25* siRNAs in the wildtype and EGFR(Y1068F) SUM159PT mutants. **(I)** Western blot showing the levels of EGFR, EGFR(Y1068) phosphorylation, TRIM25, KEAP1, NRF2, GPX4, FSP1 in SUM159PT cells with and without CAP treatment, and with and without EGFR(Y1068F) mutation. **(J)** Immunofluorescence images showing the intensity and cellular distribution of NRF2 in SUM159PT cells with and without transfection with *TRIM25* siRNAs, and with and without EGFR(Y1068F) mutation. **(K)** Immunoprecipitation results showing EGFR K48 and K63 ubiquitination in SUM159PT cells with and without transfection with *TRIM25* siRNAs in the wildtype and EGFR(Y1068F) mutant SUM159PT cells. * represents statistically significant. Significance level: * <0.1, ** <0.01, *** <0.001, ****<0.0001.

According to the mass spectrometry (MS) aiming for identifying proteins differentially interacting with the EGFR(Y1068) site, more proteins were found to interact with the EGFR(Y1068F) mutant than the wildtype SUM159PT cells (**Figure 3A**, **3B**). Specifically, 162 and 35 proteins were present and absent in the list of proteins interacting with the EGFR(Y1068F) mutant, respectively, among which TRIM25 was the sole E3 ubiquitin ligase (**Supplementary Table 1**). Immunoprecipitation results confirmed the enhanced interactions between TRIM25 and EGFR(Y1068) in EGFR(Y1068) mutants (**Figure 3C**, **3D**). In addition, TRIM25 (an E3 ubiquitin ligase of KEAP1^33^ (**Figure 3G, 3H**)) underwent K48 auto-degradation in response to CAP treatment in a dose-dependent manner (**Figure 3E**, **3F**). Thus, EGFR(Y1068F) mutation, acting as a reverse operation of CAP treatment, enhanced the stability of TRIM25 and its availability in interacting with KEAP1 (**Figure 3D**). Silencing TRIM25 did not vary the level of EGFR but enhanced KEAP1 expression and reduced levels of NRF2 and GPX4 (**Figure 3G**), indicating that TRIM25 was an upstream player of KEAP1, NRF2 and GPX4. In addition, Silencing *TRIM25* reduced K48 ubiquitination of KEAP1 in both cells harboring the wildtype EGFR and EGFR(Y1068F) mutation (**Figure 3H**), suggesting that TRIM25 was a downstream player following EGFR(Y1069) activation. CAP activated EGFR(Y1068), suppressed TRIM25, elevated KEAP1, reduced NRF2 and GPX4 but did not vary the level of FSP1; and EGFR(Y1068F) mutation reversed the expression profiles of these proteins in response to CAP except for FSP1 (**Figure 3I**). These results are suggestive of the upstream role of EGFR(Y1068) in the identified TRIM25-KEAP1-NRF2-GPX4 axis. Also, silencing *TRIM25* or mutating *EGFR(Y1068)* reduced the cytoplasmic distribution of NRF2, confirming the mediatory role of TRIM25 and EGFR(Y1068) in NRF2 cellular localization (**Figure 3J**). Lastly, silencing *TRIM25* did not affect EGFR K48 ubiquitination but enhanced EGFR K63 ubiquitination (**Figure 3K**), where EGFR K63 ubiquitination was required for EGFR degradation^34^. These results are suggestive of the stabilizing role of TRIM25 on EGFR via interacting with EGFR(Y1068) (**Supplementary Table 1**).

### EGFR(Y1068) mediates EGFR-p38-NRF2-GPX4 signaling

**Figure 4:**
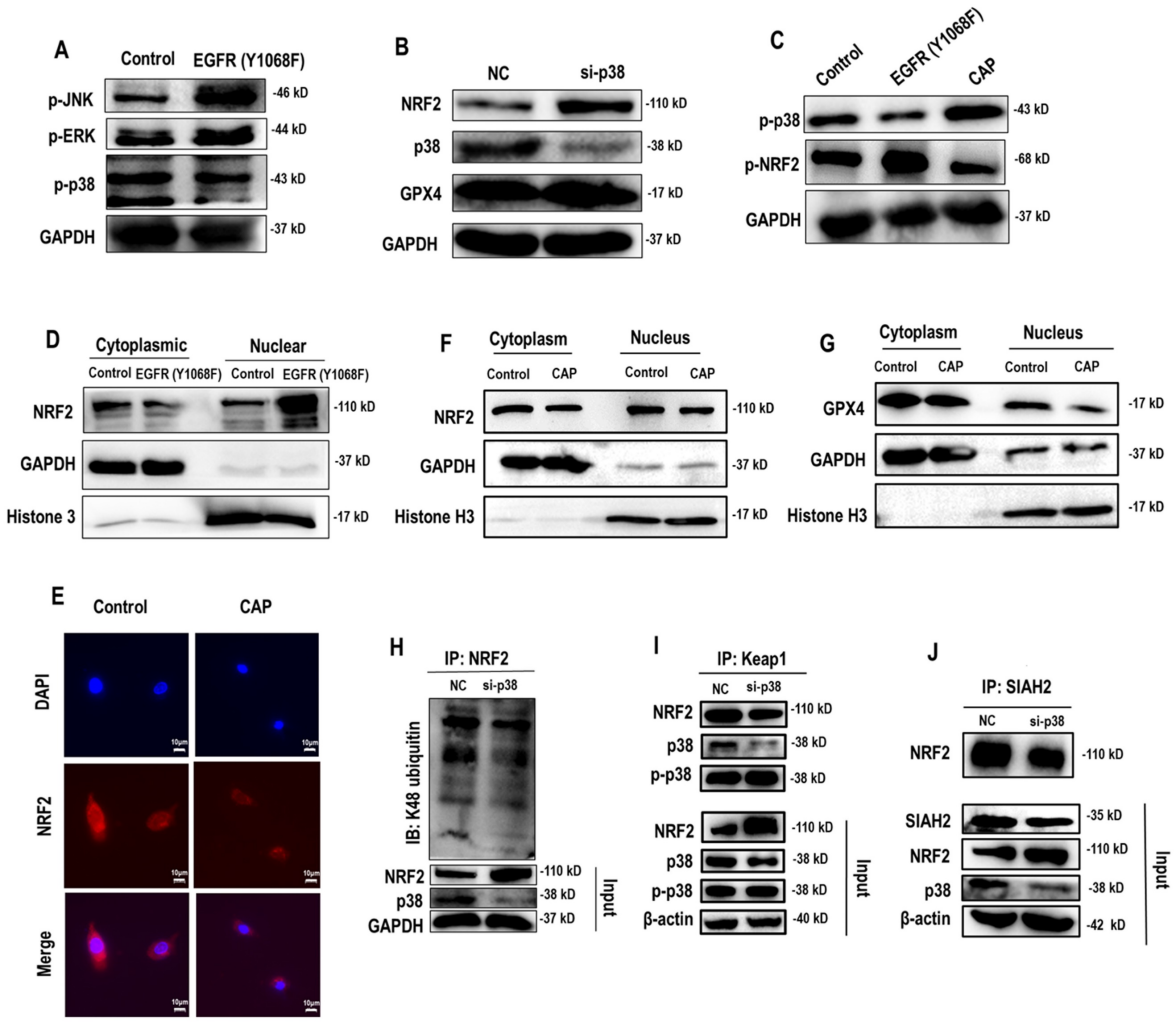
** EGFR(Y1068) mediates EGFR-p38-NRF2 signaling. (A)** Western blot showing the levels of JNK phosphorylation, ERK phosphorylation and p38 phosphorylation in SUM159PT cells with and without EGFR(Y1068F) mutation. **(B)** Western blot showing the protein expression of NRF2, p38, GPX4 in SUM159PT cells with and without transfection with *p38* siRNAs. **(C)** Western blot showing the protein expression of p38 phosphorylation and NRF2 phosphorylation status in SUM159PT cells with and without EGFR(Y1068F) mutation and with and without CAP treatment.** (D)** Western blot showing the protein level of NRF2 in the cytoplasm and nucleus of SUM159PT cells with and without EGFR(Y1068F) mutation.** (E)** Immunofluorescence images showing cellular distribution of NRF2 in SUM159PT cells with and without CAP treatment.** (F)** Western blot showing the protein level of NRF2 in the cytoplasm and nucleus of SUM159PT cells with and without CAP treatment. **(G)** Western blot showing the protein level of GPX4 in the cytoplasm and nucleus of SUM159PT cells with and without CAP treatment.** (H)** Immunoprecipitation results showing EGFR K48 ubiquitination in SUM159PT cells with and without transfection with *p38* siRNAs. **(I)** Immunoprecipitation results showing interactions between KEAP1 and NRF2, p38, phosphorylated p38 in SUM159PT cells with and without transfection with *p38* siRNAs. **(J)** Immunoprecipitation results showing interactions between SIAH2 and NRF2 in SUM159PT cells with and without transfection with* p38* siRNAs. * represents statistically significant. Significance level: * <0.1, ** <0.01, ***<0.001, ****<0.0001.

Following EGFR(Y1068) activation, MAPK signaling was activated (**Figure 2D**). Among the three key MAPK players (i.e., JNK, ERK, p38), p38 was the sole protein suppressed on EGFR(Y1068F) mutation (**Figure 4A**). Importantly, silencing *p38* enhanced NRF2 and, consequently, GPX4 expression (**Figure 4B**); and EGFR(Y1068F) mutation reversed the effect of CAP in profiling phosphorylated p38 and NRF2 (**Figure 4C**). In addition, NRF2 was accumulated in the nucleus when EGFR(Y1068) was mutated (**Figure 4D**) and allocated in the cytoplasm on CAP exposure (**Figure 4E**). CAP reduced the nucleus level of both NRF2 and GPX4 without affecting their cytoplasm amount (**Figure 4F**, **4G**). Silencing *p38* reduced NRF2 K48 ubiquitination (**Figure 4H**) but enhanced the interaction between NRF2 and KEAP1 (**Figure 4I**), implicating the existence of another E3 ubiquitin ligase that mediated the degradation of NRF2 and its competition with KEAP1 in binding with NRF2. Indeed, SIAH2 is a known E3 ligase of both NRF2 and GPX4^35^. Silencing* p38* reduced the interaction between NRF2 and SIAH2 (**Figure 4J**). These collectively suggested the existence and role of the EGFR-p38-NRF2-GPX4 axis in potentiating CAP-induced ferroptosis. That is, CAP activated p38 via triggering EGFR(Y1068) phosphorylation; activated p38 led to NRF2 accumulation in the cytoplasm and promoted its K48 ubiquitination; p38-mediated NRF2 degradation was catalyzed by SIAH2 that competed with KEAP1 for binding with NRF2.

### NRF2 regulates GPX4 at both transcriptional and translational levels

**Figure 5:**
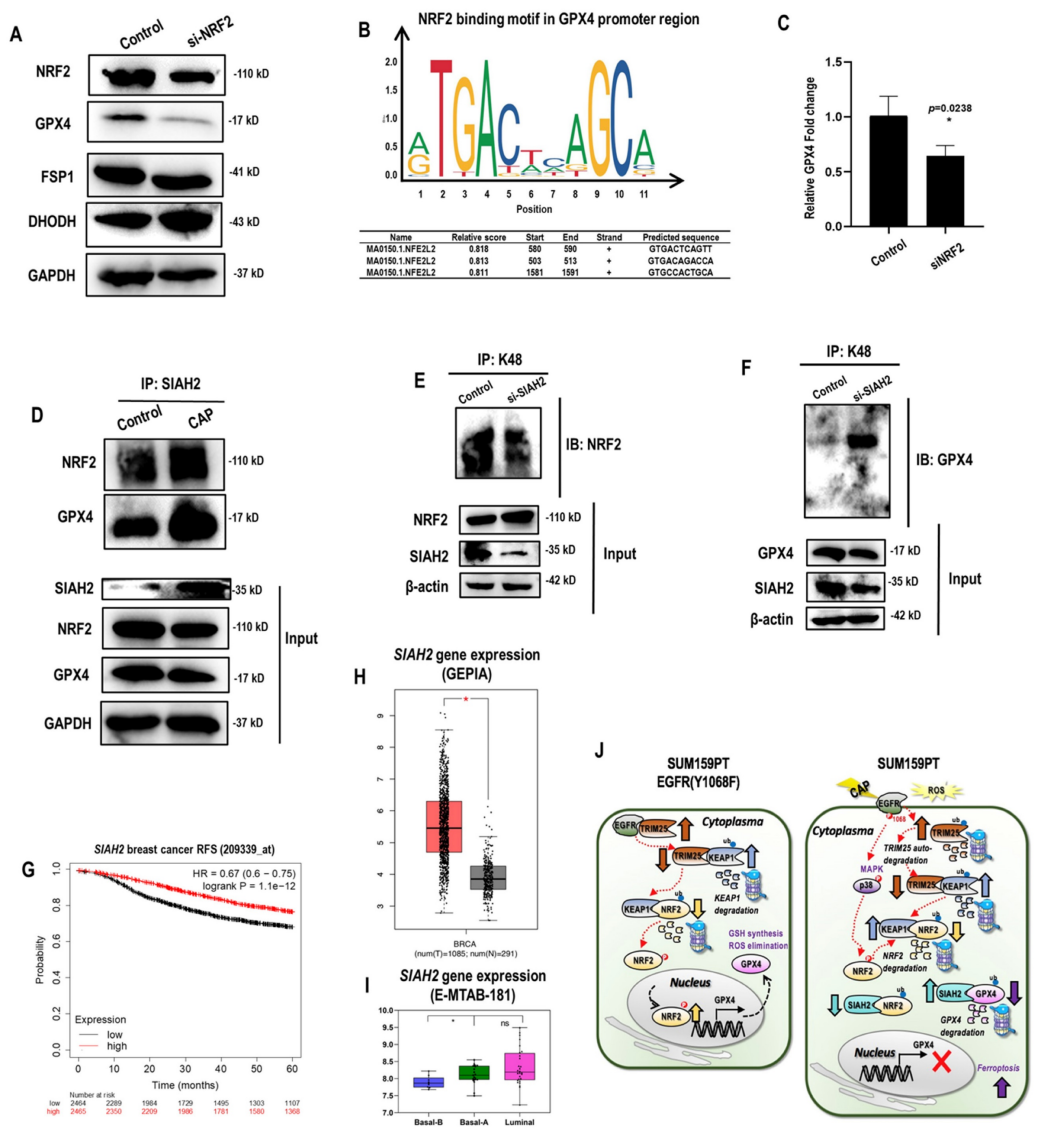
** NRF2 regulates GPX4 at both transcriptional and translational levels. (A)** Western blot showing the protein level of NRF2, GPX4, FSP1, DHODH in SUM159PT cells transfected with NRF2 siRNAs.** (B)** NRF2 transcription factor binding sites in GPX4 predicted from ConTra V3^36^. **(C)** Chromatin immunoprecipitation results showing the binding of NRF2 to the promoter region of GPX4.** (D)** Immunoprecipitation results showing interactions between SIAH2 and GPX4, NRF2 in SUM159PT cells with and without CAP treatment.** (E)** Immunoprecipitation results showing NRF2 K48 ubiquitination in SUM159PT cells transfected with *SIAH2* siRNAs.** (F)** Immunoprecipitation results showing GPX4 K48 ubiquitination in SUM159PT cells transfected with *SIAH2* siRNAs.** (G)** Breast cancer relapse free survival as stratified by *SIAH2* gene expression using probe 209339 as analyzed using gene chip data stored in Kaplan-Meier Plotter^73^. **(H)** *SIAH2* gene expression profile in breast cancer patients and normal individuals as analyzed using GEPIA 74.** (I)** *SIAH2* gene expression profile across 56 breast cancer cell lines stored in E-MTAB-181 from ArrayExpress (http://www.ebi.ac.uk/arrayexpress).** (J)** Illustrative diagram summarizing the mechanism of CAP-triggered ferroptosis in SUM159PT cells. In SUM159PT cells, CAP induces EGFR phosphorylation at Y1068 that triggers K48 ubiquitin-mediated TRIM25 auto-degradation; decreased level of TRIM25 leads to KEAP1 elevation given that TRIM25 is the E3 ubiquitin ligase of KEAP1; KEAP1 is an E3 ubiquitin ligase of NRF2, the increased level of which promotes NRF2 degradation. SIAH2 is an E3 ubiquitin ligase of both NRF2 and GPX4, where the binding affinity of SIAH2 to GPX4 is higher than that to NRF2. That is, GPX4 outcompetes NRF2 in binding to SIAH2 under SIAH2 shortage. Under sufficient SIAH2 supply in response to CAP, reduced NRF2 as a result of SIAH2- and/or KEAP2-mediated ubiquitination leads to reduced GPX4 transcription and enhanced GPX4 K48 ubiquitination. On the other hand, CAP induces EGFR phosphorylation at Y1068 that triggers p38 phosphorylation followed by retarded NRF2 phosphorylation, and this enables more cellular distribution of NRF2 and consequently less NRF2 for *GPX4* transcription. * represents statistically significant. Significance level: * <0.1, ** <0.01, ***<0.001, ****<0.0001.

Among the three ferroptosis markers that represent distinct mechanisms, GPX4 level was reduced when *NRF2* was silenced, suggesting that it was one possible downstream target of NRF2 (**Figure 5A**). Consistent with this, NRF2 was identified as the transcriptional factor of GPX4 but not that of FSP1 nor dihydroorotate dehydrogenase (DHODH) according to the relative binding scores computed from ConTra V3^36^ (**Figure 5B**). Such a physical binding was further confirmed using chromatin immunoprecipitation (ChIP), where the relative fold change of *GPX4* expression was significantly reduced to 60% of the control when *NRF2* was silenced (*p*=0.02) (**Figure 5C**). SIAH2 was proposed as the E3 ubiquitin ligase of both NRF2 and GPX4^35^, ^37^. Both the interactions of SIAH2 with NRF2 and GPX4 were increased in response to CAP treatment (**Figure 5D**), contributing to the observation that CAP suppressed both NRF2 and GPX4 (**Figure 3I**). However, silencing *SIAH2* decreased NRF2 K48 ubiquitination (**Figure 5E**, **Supplementary Figure 4A**) but increased that of GPX4 (**Figure 3F**, **Supplementary Figure 4B**). These results are suggestive of a competition between NRF2 and GPX4 under SIAH2 shortage, as well as a higher affinity of GPX4 than NRF2 in binding with SIAH2. Consistent with this, we observed enhanced SIAH2 under CAP treatment (input, **Figure 5D**). This indicates a sufficient SIAH2 production in response to CAP exposure to enable the K48 ubiquitination of both GPX4 and NRF2. Indeed, high *SIAH2* gene expression was associated with favorable relapse free survival (RFS) among breast cancer patients (**Figure 5G**) and, in particular, among patients lacking sufficient expression of ER and HER2 (including TNBCs) (**Supplementary Figure 4C**). Concordant with this,* SIAH2* gene expression was higher in breast cancer tissues than normal tissues (**Figure 5H**), and higher in basal (corresponding to TNBC cells) than luminal breast cancer cells (**Figure 5I**).

Thus far, we characterized two pathways contributing to CAP-enabled TNBC cell ferroptosis. First, CAP induced EGFR(Y1068) phosphorylation that triggered TRIM25 auto- K48 ubiquitination. As TRIM25 is an E3 ubiquitin ligase of KEAP1, decreased TRIM25 level resulted in KEAP1 up-regulation. As an E3 ubiquitin ligase of NRF2, increased level of KEAP1 led to promoted NRF2 degradation. In addition, SIAH2 is another E3 ubiquitin ligase of NRF2 that mediates the K48 ubiquitination of GPX4, with its binding affinity with GPX4 being higher than that with NRF2. Thus, SIAH2 mediated the degradation of both GPX4 and NRF2 under sufficient SIAH2 supply, yet GPX4 outcompeted NRF2 in binding with SIAH2 under SIAH2 shortage. On CAP exposure, SIAH2 was elevated that resulted in deceased protein expression of both NRF2 and GPX4. Taken together, the EGFR(Y1068)-TRIM25-KEAP1/SIAH2-NRF2 axis explained the regulatory role of CAP on the stability of GPX4. Second, CAP induced EGFR(Y1068) phosphorylation that triggered p38 phosphorylation followed by reduced NRF2 phosphorylation, and this enabled less nucleus distribution of NRF2. NRF2 is a transcription factor of GPX4 with a promotive role in gene expression. Thus, the EGFR(Y1068)-p38-NRF2 axis explained the regulatory role of CAP on the transcriptional level of GPX4 (**Figure 5J**).

### CAP creates synergies with sorafenib in triggering the ferroptosis of TNBC cells

**Figure 6:**
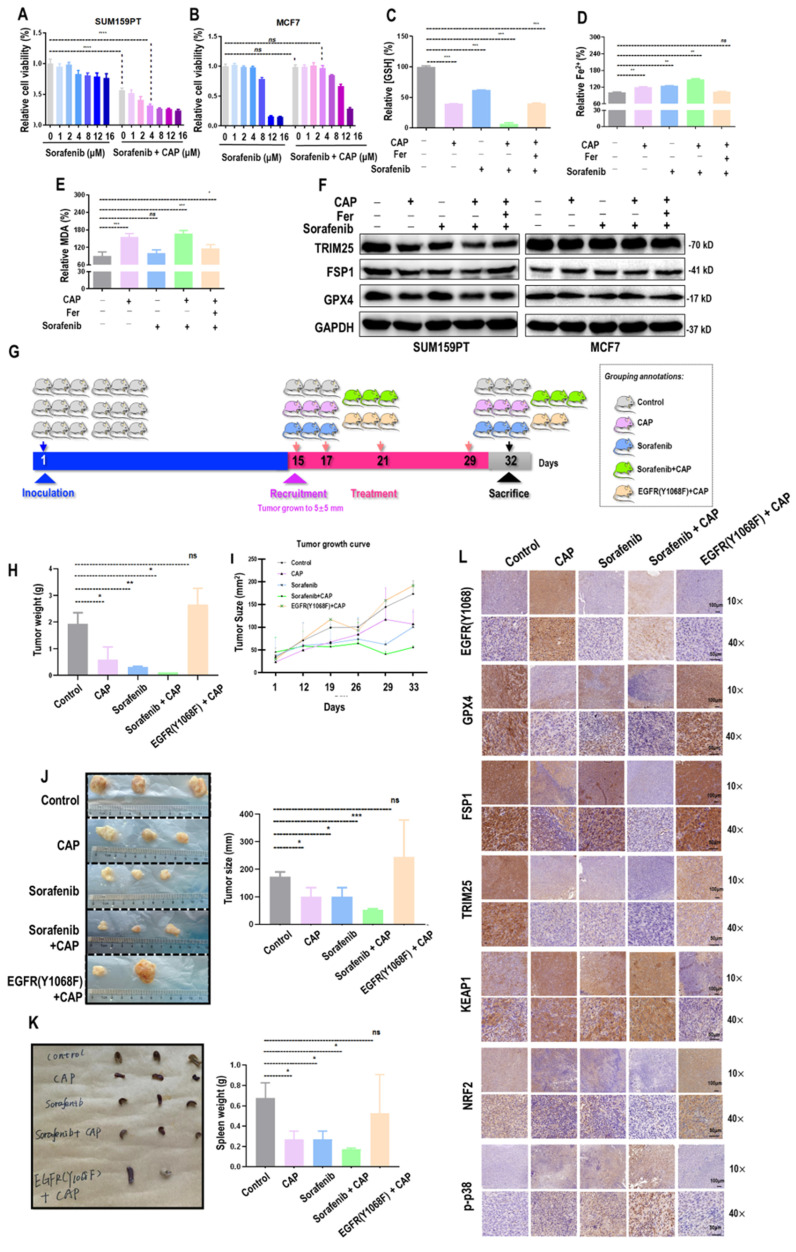
** CAP creates synergies with sorafenib in triggering ferroptosis among TNBC cells.** Relative viabilities of **(A)** SUM159PT cells and **(B)** MCF7 cells in response to sorafenib and combined use of CAP and sorafenib.** (C)** Relative GSH percentage,** (D)** relative Fe^2+^ percentage,** (E)** relative MDA percentage in SUM159PT cells receiving CAP treatment, sorafenib, 'CAP plus sorafenib', and 'CAP plus sorafenib and ferroptosis inhibitor'. **(F)** Protein levels of TRIM25, GPX4, FSP1 in SUM159PT and MCF7 cells receiving CAP treatment, sorafenib, 'CAP plus sorafenib', and 'CAP plus sorafenib and ferroptosis inhibitor'. **(G)** Illustrative diagram showing animal assay design.** (H)** Tumor weight, **(I)** tumor size**, (J)** tumor images and quantifications,** (K)** spleen images of SUM159PT-inoculated mice receiving CAP treatment, sorafenib, 'CAP plus sorafenib', and mice carrying SUM159PT EGFR(Y1068F) mutants in response to CAP treatment.** (L)** Immunohistochemistry staining of EGFR(Y1068), GPX4, FSP1, DHODH, TRIM25, KEAP1, NRF2, p38 phosphorylation in SUM159PT-inoculated mice receiving CAP treatment, sorafenib, 'CAP plus sorafenib', and mice carrying SUM159PT EGFR(Y1068F) mutation in response to CAP treatment. * represents statistical significance. Significance level: * <0.1, ** <0.01, ***<0.001, ****<0.0001.

Sorafenib is a known targeted therapy capable of inducing ferroptosis across a wide spectrum of cancer cells^38^. We explored the feasibility of enhancing the efficacy of CAP by synergizing it with sorafenib. The viability of SUM159PT cells was significantly reduced after CAP treatment (60% of the untreated group, *p*<E-4) that was further reduced in a dose-dependent manner when CAP was combinatorially used with sorafenib (**Figure 6A**). The plateau of cell viability was reached at 4
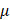
M of sorafenib, and effective reduction on cancer cell viability was observed when CAP was used together with 4
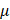
M sorafenib (**Figure 6A**). Yet, no significant alteration was observed in MCF7 cells when 4
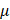
M sorafenib was used alone or in couple with CAP (**Figure 6B**). The synergistic effect between CAP and sorafenib in inducing SUM159PT cell ferroptosis was demonstrated using canonical indexes of ferroptosis including relative GSH percentage (*p*=3E-4, **Figure 6C**), Fe^2+^ percentage (*p*=7.4E-3, **Figure 6D**), and MDA percentage (**Figure 6E**). The protein levels of key ferroptosis markers GPX4 and FSP1, as well as TRIM25 were the lowest in SUM159PT cells receiving both CAP and sorafenib (**Figure 6F**). However, the profiles of these proteins stayed invariant in MCF7 cells (**Figure 6F**).

Three mice were recruited in each designed group, and one mouse from the 'EGFR(Y1068F)+CAP' group died by the end of this study (**Figure 6G**). The averaged weight of tumors dropped to 1/3 of the control when receiving CAP treatment and to approximately 1/10 of the control in the 'sorafenib+CAP' group; EGFR(Y1068F) mutation reduced the sensitivity of TNBCs to CAP treatment (**Figure 6H**). CAP and sorafenib showed similar efficacies in reducing the tumor size, combining CAP and sorafenib reached the best efficacy in controlling tumor growth, and treating tumors harboring the EGFR(Y1068F) mutation with CAP showed the same therapeutic outcome as the control (**Figure 6I, 6J**). CAP, sorafenib and their synergized treatment all reduced the size of mice spleen, and EGFR(Y1068F) mutation rewired such an effect (**Figure 6K**). Immunohistochemistry staining revealed reduced intensities of GPX4, TRIM25, NRF2, and elevated levels of EGFR(Y1068), phosphorylated p38, KEAP1 in mice samples receiving CAP, sorafenib, 'sorafenib+CAP' treatments as compared with the untreated group, with the largest difference being observed between the 'sorafenib+CAP' group and the control (**Figure 6L**). EGFR(Y1068F) mutation rendered TNBCs immune to CAP treatment given the similar intensities observed between the control and the 'EGFR(Y1068F)+CAP' group regarding the staining profiles of these ferroptosis-related protein markers (**Figure 6L**).

## Discussion

There were at least exist three independent pathways contributing to ferroptosis, with featured proteins being GPX4, FSP1 and DHODH, respectively (**Supplementary Figure 5A**). By converting glutathione from the reductive (GSH) to the oxidative (GSSG) state in the cytoplasm and mitochondria, GPX4 protects cells from lipid peroxidation with the aid of Fe^2+^^39^ (**Supplementary Figure 5A**). FSP1 suppresses PL-OO• formation by oxidizing ubiquinol (CoQH_2_) to ubiquinone (CoQ)^29^; and DHODH operates in the inner mitochondria membrane and potentiates ferroptosis by reducing CoQ to CoQH_2_^40^ (**Supplementary Figure 5A**). Jo *et al.* recently reported CAP-induced ferroptosis in human lung cancer cells via suppressing FSP1 but not GPX4^41^ (**Supplementary Figure 5A**). CAP has been reported capable of depleting FSP1 in human lung cancer cells^41^. Consistent with this, we observed the suppressive role of CAP on FSP1 in TNBC cells (**Figure 1I**). Importantly, we reported the sabotaging role of CAP on redox homeostasis in TNBC cells towards an environment favorable for ferroptosis via two EGFR(Y1068)-centered molecular axes, i.e., EGFR(Y1068)-KEAP1/SIAH2-NRF2 and EGFR(Y1068)-p38-NRF2, which synergistically drove GPX4-dependent ferroptosis. Therefore, the potentiating role of CAP on cancer cell ferroptosis may concomitantly involve multiple mechanisms, the specific signaling axes involved are collectively determined by cell intrinsic features such as the redox status and external perturbations such as environment stimuli in addition to CAP. Mechanically, these different ferroptosis axes may take on the action under different dosages of free radicals or be controlled by different leading components of CAP.

We hypothesized the leading role of OH•, an intrinsic component of CAP and a primary source for potentiating lipid oxidation (**Supplementary Figure 5A**), in triggering TNBC cell ferroptosis. The results showed that quenching OH• restored cells' viabilities from approximately 30% to 65% after CAP treatment (**Figure 1L**). Thus, reactive species other than OH• may collectively explain about 35% of the CAP-induced cancer cell ferroptosis, which is equivalent to that of OH•. In other words, OH• plays a dominant role in potentiating CAP-induced TNBC cell ferroptosis by contributing to around half of the observed ferroptotic effects.

Hydroxyl radical can oxidize polyunsaturated fatty acids (PUFA), the level of which controls the sensitivity of cells to redox modulatory tools including CAP. Alkylglycerone phosphate synthase (AGPS), long-chain acyl-CoA synthetase 1 and 4 (ACSL1, ACSL4) are critical enzymes controlling the synthesis of PUFA-CoA with known roles in shaping the sensitivity of cells to ferroptosis^42^-^44^ (**Supplementary Figure 5A**). Genes encoding these enzymes are all over-represented in TNBC cells (**Supplementary Figure 5B-5D**), explaining, at least partially, the selectivity of TNBC cells to CAP treatment (**Figure 1A**), and the cancer spectrum feasible for receiving CAP treatment.

Importantly, ferroptosis has been largely considered to be dependent on the Fenton reaction (Fe^2+^ + H_2_O_2_ → Fe^3+^ + OH• + OH^-^). The Fenton reaction, on the other hand, has been indicated to drive nucleotide and ATP syntheses in cancer, and thus contributes to cancer cell division and stemness^45^. Thereby, the inductive role of CAP on ferroptosis among TNBC cells is in line with its recently identified efficacy in retarding cancer stemness^14^; and the higher vulnerability of TNBC cells in response to CAP treatment than the other subtypes such as luminal cells can be attributed to the higher percentages of cancer stem cells possessed by TNBC cells.

Ferroptosis cannot explain all CAP-triggered cancer cell death, with around 20% death events being not rescuable by ferroptosis inhibitors (**Figure 1C**). This suggests the co-existence of other programmed death events induced by CAP. Besides apoptosis^46^, necroptosis^47^, autophagic cell death^48^ and immunogenic cell death^49^ that have been previously reported, we forecast the role of CAP in potentiating cuproptosis and reactions alike. Specifically, multivalent metals and radicals can react to recycle valence state of multivalent metals towards ROS production via activating H_2_O_2_ taking advantages of the dual roles of H_2_O_2_ played in redox catalysis (i.e., both reductant and oxidant roles). This implies the potentiating role of CAP on cuproptosis besides ferroptosis and, creatively, the existence and triggering of other innovative forms of death programs potentiable by multivalent metals such as manganese (Mn^4+^/Mn^2+^), chromium (Cr^6+^/Cr^3+^), cobalt (Co^3+^/Co^2+^), vanadium (V^5+^/V^3-^), and molybdenum (Mo^6+^/Mo^4+^)^50^. Besides having multiple valent states, these elements are all micronutrients naturally existing in the human body, making it possible to cause cell death on ROS trigger. Besides Fenton-like reactions, disulfidptosis may also occur on CAP induction. This is because of the role of ROS in inducing disulfide stress and thus abnormal intracellular disulfide bonds, the atypical formation of which is characterized by disulfidptosis^51^.

EGFR alterations have been implicated in the carcinogenesis of many malignancies and thus been considered as a well-established onco-therapeutic target of a plethora of cancers such as colorectal cancer, non-small cell lung cancer, as well as head and neck squamous cell carcinoma^52^. Several monoclonal antibodies such as cetuximab, panitumumab, nimotuzumab, necitumumab have already been examined in clinical trials^53^. Yet, severe side effects such as diarrhea, mouth sores, loss of appetite and skin problems were reported for targeting EGFR besides drug resistance given the critical roles EGFR played in cells under both pathological and physiological conditions^54^. EGFR tyrosine kinase inhibitors, though may release these adverse syndromes by directly and solely suppressing EGFR intracellular protein tyrosine kinase, generated insignificant response rates in, e.g., breast cancers^55^. Suppressed EGFR has been implicated to protect non-small-cell lung tumor cells from ferroptosis^56^. Here, we showed that, instead of inhibiting EGFR or EGFR phosphorylation, CAP selectively turned on the ferroptotic program of cancer cells by potentiating EGFR phosphorylation at Y1068 and, concomitantly, enhancing cellular redox level. CAP may represent an opposite strategy to conventional approaches relying on EGFR, which fosters a favorable environment for ferroptosis by activating EGFR(Y1068).

Interestingly, CAP enhanced EGFR K63 but not K48 ubiquitination in TNBC cells, where EGFR(Y1068) activation played an essential role (**Figure 3K**). K63 ubiquitination is required for EGFR degradation^34^. It is known that EGFR activation and ubiquitination are coupled events in healthy but not malignant cells^57^, where the EGFR(Y1068) site plays a connective role and is necessary for EGFR ubiquitination^58^. Thus, by activating EGFR(Y1068), CAP restored the ratio between the levels of total and activated EGFR back to that at the healthy state, which may have profound physiological implications and deserve more focused investigations. In addition, K63 ubiquitination can drive NF
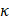
B and MAPK-mediated immune signaling^59^, suggesting the relevance of CAP in modulating the immune response of cancer cells and a novel avenue for CAP-enabled immunology.

EGFR over-representation has been reported to occupy up to 78% TNBCs^52^, ^60^-^62^. This, on one hand, explains the higher sensitivity of TNBC cells to CAP treatment than non-TNBC cells and, on the other hand, suggests the possibility of using EGFR in diagnosing the sensitivity of breast cancer patients to CAP treatment. However, CAP cannot be viewed as a targeted therapy recognizing EGFR, as CAP targets the system controlling cell redox homeostasis where the targets may dynamically vary with cellular redox status and differ among different types of cells. Thus, whether the therapeutic and diagnostic values of CAP rested on EGFR uncovered here are extensible to other cancer types requires intensive pre-clinical validation and clinical evidence.

Using TNBC as the tumor model, we characterized the role of EGFR in sensitizing cancer cells to CAP treatment. This does not exclude the possible involvement of other receptors in CAP-induced signaling^63^, which is not covered by this study. Thus, pre-examining the response pattern of a given cancer type under a certain context is of the fundamental importance in translating CAP into the clinics as a precision onco-therapeutics.

We identified two signaling axes in response to CAP that were both initiated from EGFR(Y1068) activation and ended with GPX4 inhibition, which were the canonical (i.e., p38-NRF2-GPX4) and the *de novo* (i.e., TRIM25-KEAP1/SIAH2-NRF2-GPX4) paths. These two paths may concomitantly take actions or function as the alternative of the other under certain circumstances. These two axes here do not exclude the possible existence of other signaling paths, taking into account the multimodal nature of CAP and complications of the network relaying CAP-imposed perturbations.

The inductive role of CAP on ferroptosis is not limited to TNBCs, and has been reported applicable to other transformed cells such as skin cancers^64^, non-small cell lung cancers^41^, ^65^, and colorectal cancers^66^. Besides being the first to document CAP-triggered ferroptosis of TNBC cells, this study for the first time associated EGFR(Y1068) with cancer cell ferroptosis. Actually, EGFR(Y1068) not only mediated the ferroptosis of cancer cells in response to CAP treatment but also other cell death programs such as apoptosis^46^ and autophagy^10^. This implicated the potentiating role of EGFR and, in particular, EGFR(Y1068) in CAP-primed cancer cell death as well as the diagnostic and therapeutic values of this phosphorylation site. This makes it possible to screen cancers feasible for receiving CAP treatment and enables the design of innovative therapeutics via stimulating EGFR(Y1068) phosphorylation instead of blocking the whole EGFR receptor for treating cancers with little adverse effect.

As a promising treatment modality for potentiating ferroptosis among TNBCs, we observed synergies between CAP and sorafenib (an anti-cancer drug capable of inducing ferroptosis) in treating TNBC cells. Actually, strategies relying on the trigger of ferroptosis such as the use of exosome-encapsulated erastin have already been proposed for treating cancers^39^, ^67^, ^68^. Yet, CAP is unique in being capable of specifically killing cancer cells and is characteristic of the multi-modality nature that does not rely on any single mechanism in arresting cell malignancy^18^. Though complicating our understandings on the anti-cancer molecular mechanisms of CAP, these traits may render CAP a promising onco-therapeutic modality with little likelihood of developing adverse effects and drug resistance.

CAP can be applied in the clinics as a possible cancer-ablation tool through direct ejection using CAP generation sources, or through preparing plasma-activated medium (PAM) followed by PAM transfusion or perfusion. PAM can be prepared using various liquids typically used in the bed side such as the Ringer's lactate solution and physiological saline solution^69^. Though the launch of CAP seems to be easy, there exist several hurdles hindering the clinical translation of CAP in the field of oncology. First of all is the transient life spans of the short-lived reactive species within CAP including the leading component OH•^14^ that lasts 10^-9^s *in vitro* and *in vivo*^70^. Although the activities of CAP can be preserved to some extent in the form of liquid, PAM contains largely long-lived species^71^, ^72^, rendering its anti-cancer efficacy as less competent as that of CAP. Also, the limited penetration in-depth of CAP further restricted the clinical scenarios feasible for receiving CAP therapy, and made its treatment on visceral tumors largely rely on surgery-assisted irradiation. Resolving these challenges may require the aid of cross-disciplinary techniques such as nanomaterials that is beyond the scope of this study but deserves intensive explorations.

## Conclusion

Our study, for the first time, reported CAP-induced ferroptosis of TNBC cells and the regulatory role of EGFR(Y1068) in this process. By suppressing the GPX4 anti-oxidant system via the EGFR(Y1068)-TRIM25-KEAP1/SIAH2-NRF2-GPX4 and EGFR(Y1068)-p38-NRF2-GPX4 axes, and imposing additional cellular redox stress, CAP specifically turned on the ferroptotic program of TNBC cells; and the efficacy was further enhanced by being synergistically used with sorafenib. The uncovered mechanism not only substantiated our understandings of the signaling network potentiating the specificity of CAP against malignant cells, but also underpinned the mediatory roles of EGFR in delivering the therapeutic advantages of CAP. Specifically, while preserving a similar treatment efficacy, CAP circumvented the side effects accompanied with EGFR inhibitors by activating EGFR that rendered cancer cells vulnerable to ferroptosis. Also, this study suggested the diagnostic use of EGFR(Y1068) phosphorylation in screening cancers sensitive to CAP treatment and brought hope for cancer patients harboring the intact EGFR(Y1068) site.

## Materials and Methods

### Cell culture

Five cell lines were used in this study, including four TNBC cells (SUM159PT, MDAMB231, MDAMB468, HCC1937) and one non-TNBC line (i.e., MCF7). All cells were available from American Type Culture Collection (Manassas, VA, USA), purchased from Nanjing Kemo biomedical Co., Ltd., and authenticated using Short Tandem Repeat (STR) analysis following the instructions described in 2012 in ANSI Standard (ASN-0002). SUM159PT cells were cultured in F12 (#P2090359, Damas) supplemented with 5% FBS (#FSP500, ExCellBio), 0.325% insulin, 1% HEPES, 0.0276% hydrocortisone and 1% penicillin-streptomycin (#BL505A, Biosharp), and the rest cell lines were cultured in DMEM (#RNBK4719, SIGMA) supplemented with 10% FBS and 1% penicillin-streptomycin. All cells were cultured under 37°C, 5% CO_2_.

### CAP and plasma-activated medium (PAM) generation

A home-made CAP source was used in this study which is composed of a controlled power supply, helium (He) gas cylinder, rotameter and plasma jet (**Supplementary Figure 1**). The peak to peak voltage applied to the electrode was set in the range of 0.96 KV to 1.24 KV, the sine wave frequency was set at 10 kHz, the flow rate of He was set at 1 L/min, and the distance between the CAP source and the dielectric surface was fixed to 13 mm. PAM was prepared by setting the distance between the CAP nozzle and the media surface to 13 mm, the peak-to-peak electrode voltage was set to 1.1 KV, the sine wave frequency was set to 8.8 KHz, the He gas flow rate was set to 1 L/min. In an assay, 2 mL of the cell culture medium in a 12-well plate was exposed to CAP for 4 min. The CAP-treated group was obtained by replacing cell culturing medium with PAM.

### Reagents and antibodies

Primary antibodies: NRF2 (#16396-1-AP, Proteintech, 1:1000), TRIM25 (#ab167154, Abcam, 1:1000), GPX4 (#ab125066, Abcam, 1:1000), DHODH (#14877-1-AP, Proteintech, 1:2000), FSP1 (#20886-1-AP, Proteintech, 1:1000), KEPA1 (#10503-2-AP, Proteintech, 1:1000), EGFR (#2085S, CST, 1:1000), EGFR (Tyr1068, #3777S; Tyr1086, #2220S; Tyr1148, #4404S; Tyr992, #2235S; CST, 1:1000), p-ERK (#4370s, CST, 1:2000), p-JNK (#4668s, CST, 1:1000), p38 (#8690s, CST, 1:1000), p-p38 (#4511s, CST, 1:1000), K48/K63 (#8081s/5621s, CST, 1:1000), SIAH2 (#12651-1-AP, Proteintech, 1:1000), β-actin (#PTM-5018, PTM-BIO, 1:1000), GAPDH (#AC001, ABclonal, 1:1000), Histone-H3 (#ab176842, Abcam, 1:1000).

Secondary antibodies: HRP labeled Goat anti Rabbit IgG (#A0208, Beyotime, 1:5000), HRP labeled Goat anti Mouse IgG (#A0216, Beyotime, 1:5000).

### Plasmid and construction of cells carrying stable EGFR (Y1068F) mutation

The plasmid pRP[CRISPR]-EGFP/Puro-hCas9-U6> {mEGFR[gRNA]} and Single-stranded Oligo Donor (ssODN)-mEGFR[NG_007726.3](Y108F) (**Supplementary Figure 3A**) were designed and purchased from Vector Builder (China).

The RP[CRISPR]-EGFP/Puro-hCas9-U6 plasmids were isolated using the FastPure Plasmid Mini Kit (#DC201-01, Vazyme). The ssODN was ethanol-precipitated, re-suspended (100 μM) using 10 mM Tris-HCl (pH=7.5), and diluted to 1 μM prior to transfection.

The limited dilution approach was used to isolate monoclonal cells. When cells grew to approximately 50%-70% confluence in 6-well plates, they were supplemented with 1 μM ssODN, 1 μg pRP[CRISPR]-EGFP/Puro-hCas9-U6 and 6 μL lipofectamine 3000 (#L3000008, Invitrogen) and incubated for 8 h. The medium was refreshed with the medium supplemented with 2 μg/mL puromycin (#58-58-2, Beyotime) 72 h after transfection. The medium was refreshed every 2 to 5 days until one cell was left in a single well. Cells were successively cultivated under 2 μg/mL puromycin selection until EGFR(Y1068F) mutation was stable (**Supplementary Figure 3B**).

### Transfection

Cells were seeded in 6-well plates, and siRNA was dissolved in diethypyrocarbonate (DEPC)-treated water. 200
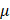
L jetbuffer (#B210610, Polyplus), 5 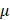
L siRNA, 10 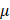
L jetprimer (#0000000373, Polyplus) were subsequently added to each well. The cell culture medium was updated 6 h after the transfection reagent was supplemented, and cultured for 24-48 h prior to the following steps. The siRNA sequences were designed using primer blast (https://www.ncbi.nlm.nih.gov/tools/primer-blast/) and purchased from GENCEFE (Wuxi, China).

### Cell proliferation assay

The GSH and GSSG detection kit (#S0053, Beyotime) was used to perform the cell proliferation assay. Appropriate amount of cells were inoculated in 96-well plates followed by incubation overnight. Cells were treated and cultured for 24 h. The MTT treatment solution was prepared according to the ratio of medium: MTT = 1:9. 500
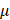
L MTT treatment solution and 100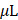 DMSO were added to each well. The mixture was incubated in the darkness. The absorbance of each well was measured and the cell viability of the experimental group was determined considering that of the control group to be 100%.

### Glutathione (GSH) assay

Appropriate amounts of cells were inoculated in 6-well plates followed by incubation overnight. Cells were treated and cultured for 24 h. After cells were collected, freshly prepared protein remover M solution was added. Samples were rapidly frozen and thawn twice using liquid nitrogen and 37°C water bath. Samples were put in a 4°C refrigerator standstill for 5 min, followed by centrifugation at 10000g for 10 min. The supernatant was collected and the total glutathione was determined. The total glutathione detection working solution was prepared following the manufacture's protocol, and the standard curve was drawn by diluting the standard sequentially according to a pre-designed concentration gradient. The sample and total glutathione detection working solution were added into a 96-well plate in order, mixed well, and incubated at the room temperature for 5 min. 50 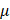
L of 0.5mg/mL NADPH solution was added and mixed well. The total amount of glutathione in each well was determined according to the absorbance obtained from the microplateReader (#Epoch, BioTek) and the standard curve.

### MDA, Fe^2+^ assay

Appropriate amounts of cells were inoculated in 6-well plates followed by incubation overnight. Cells were treated and cultured for 24 h. The lysis solution was added and lysed cells were put on ice for 20 min. Cells were centrifuged at 12000 g for 30 min, and the supernatant was collected to prepare wells of different reactions. The absorbance was measured using the microplateReader (#Epoch, BioTek) following the manufacture's protocol of the iron kit (#I291, DOJINDO) and the Fe^2+^ amount was determined.

### ROS quantification assay

Appropriate amounts of cells were inoculated into 24-well plates pre-placed with climbing pieces for 24 h. Cells were treated and cultured for 24 h. The culture medium was discarded and replaced with 10 mM 2', 7'-dichlorofluorescin diacetate (HCFH-DA) that was diluted using fresh culture medium. Cells were cultured for 30 min at 37°C and 5% CO_2_. ROS was imaged and quantified using the microscope (#Axio Imager Z2, ZEISS).

### Lipid ROS quantification assay

Cells were seeded on 12-well plates until 80% confluence. Following the removal of the cultivating medium, PAM was added and incubated with cells for 2 h. Then, cells were treated with 2.5 μM BODIPY 581/591 C11 dye (Invitrogen, Carlsbad, USA) for 15 min in a humidified incubator, washed using 1×PBS and trypsinized to obtain a single-cell suspension. Lipid peroxidation levels were detected using S3e Cell Sorter (Bio-Rad, Hercules, California, USA).

### ROS scavenger assay

Sodium pyruvate (10 mM), uric acid (100 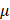
M), mannitol (200 mM), Tiron (20 mM), hemoglobin (20 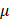
M), and monopotassium phosphate (1 mM) were used to trap H_2_O_2_, O_3_, ·OH, ·O^2-^, ·NO and e^-^, respectively, which were purchased from Sigma-Aldrich (Australia). Cells were seeded in the 96-well plate and cultured for 24 h at a concentration of 5000 cells/well. 100%PAM was prepared by treating the medium with CAP for 10 min. 90 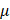
L of 100%PAM and 10
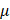
L of each type of ROS scavengers were mixed and incubated together for 1 min. 50 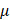
L of the mixture was added to cells for 30 s followed by immunofluorescence imaging. All scavengers were proven non-toxic at the working concentrations.

### Western blot

The total proteins were extracted from tissues and cells using RIPA (#P0013B, Beyotime) supplemented with protease and phosphatase inhibitors (#P1005, Beyotime). Concentration of the extracted proteins were determined using the BCA method (#P0010, Beyotime). Extracted proteins were separated by 10% SDS-PAGE and transferred to poly (vinylidene fluoride) (PVDF) membranes (#IPVH00010, Milipore). Membranes were blocked using the blocking buffer (5% evaporated milk) and incubated using the indicated primary antibody overnight at 4°C. Membranes were incubated using horseradish peroxidase (HRP) conjugated secondary antibodies for 1 h at the room temperature and washed using tris-buffered saline and Tween 20 (TBST). Western blots were developed using enhanced chemiluminescence (#E412-02, Vazyme).

### Co-immunoprecipitation

The IP/Co-IP kit (#abs955, Absin) was used to perform this assay. Total proteins were extracted from cells using lysis buffer supplemented with protease and phosphatase inhibitors (#P1005, Beyotime). 5% protein supernatant was left as the input. The target antibody was added to the remaining protein supernatants and incubated overnight at 4°C. 5 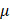
L protein A agarose and protein G agarose were added to the mixture and incubated at 4°C for 1-3 h. The mixture was centrifuged at 12000 g for 2 min. The protein agarose antigen antibody complexes were washed using the lysis buffer and resuspend using the loading buffer. The supernatant was separated by sodium dodecyl sulfate-polyacrylamide gel electrophoresis (SDS-PAGE), and protein-protein interactions were detected using western blot.

For ubiquitination, cells were pre-treated with 40 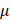
M MG132 (#HY-13259, MedChemExpress) for 2 h.

### Immunofluorescence

Appropriate amounts of cells were placed on glass coverslips overnight. Cells were treated following the experimental design, fixed using 4% paraformaldehyde, and permeabilized with 0.1% triton X-100 for 5 min. Cells were blocked with 5% bovine serum albumin for 30 min at the room temperature and incubated with the primary antibody overnight at 4°C. Cells were incubated with their respective fluorescent dye conjugated secondary antibodies for 1 h at 37°C, and stained with 4', 6-diamidino-2-phenylindole (DAPI). The staining results were observed using the upright fluorescence microscope (#Axio Imager Z2, Zeiss).

### Immunohistochemistry staining

Paraffin-embedded sections were dewaxed and rehydrated, and the activity of antigens was restored by boiling them in the citrate buffer (pH=6.0). The endogenous peroxidase activity was blocked by incubating the sections in 3% H_2_O_2_. Samples were blocked using 10% goat serum followed by incubation overnight at 4°C with primary antibodies against GPX4, FSP1, DHODH, TRIM25, KEPA1, NRF2, and EGFR(Y1068). Samples were incubated using HRP-conjugated secondary antibodies, and immunoreactive proteins were detected using 3, 3'-diaminobenzidine (DAB) staining (#CW2069s, CoWin Biosciences).

### Scanning electron microscopy

Cells were collected and fixed using 2.5% glutaraldehyde overnight, pre-stained using 1% osmic acid and placed at 4°C for 2 h. Cells were dehydrated using gradient ethanol (50%, 70%, 90%), 90% ethanol acetone mixture (90% ethanol: 90% acetone = 1:1), and 90% acetone, washed three times using 100% acetone. Cells were soaked in 1:1 acetone epoxy resin solution (acetone: epoxy resin = 1:1) for 1 h, in 1:2 acetone epoxy resin solution (acetone: epoxy resin = 1:2) overnight, in epoxy resin for 3 h and in refreshed epoxy resin for additional 4 h. Samples were polymerized at 60°C for 48 h, and spliced into 50 nm ultrathin sections using ultramicrotome (#EM UC7, Leica). Samples were stained using colloidal gold solution, and observed under biological transmission electron microscope (#Tecnai G2 Spirit Biotwin, FEI). This assay was performed at the Instrumental Analysis Center of Shanghai Jiao Tong University.

### Mass spectrometry

The total protein was extracted by RIPA lysis buffer (#P0013B, Beyotime) supplemented with protease and phosphatase inhibitors (#P1005, Beyotime). The extracted protein sample was separated by 10% SDS-PAGE followed by Coomassie brilliant blue (#P0017B, Beyotime) staining. Extracted protein samples were sent to PTM BioLab Inc. (Hangzhou, China) for mass spectrometry analysis.

### Animal experiment

All animal experiments were carried out in accordance with the Guidelines for Nursing and Utilization of Experimental Animals issued by the National Institutes of Health and approved by the Animal Experiment Center of Jiangnan University.

SUM159PT and SUM159PT EGFR(Y1068F) cells suspended in PBS were subcutaneously injected into the right forelimbs of 15 4-week-old female nude BALB/c mice. The initial weight per mouse was 21 ± 2g. Each mouse was injected with 1 × 10^6^ cells. After 2 weeks feeding, mice with tumors grown to 5 ± 0.5 mm in diameter were recruited in this study. In total, 15 mice were included and distributed into five groups, with each group having 3 mice. Specifically, 12 BALB/c mice inoculated with SUM159PT cells were randomly divided into four groups, namely 'Control' (receiving untreated medium), 'CAP' (receiving CAP), 'Sorafenib' (receiving Sorafenib) and 'Sorafenib+CAP' (receiving Sorafenib+CAP). Three BALB/c mice inoculated with SUM159PT_EGFR(Y1068F) cells were grouped and named as 'EGFR(Y1068F)+CAP'.

Mice were treated on the 1^st^, 3^rd^, 7^th^ and 15^th^ day after the recruitment. 4 μM Sorafenib was used. Sorafenib and PAM were mixed at a ratio of 1:1. During each operation, mice were pre-anesthetized intraperitoneally with ketamine (10 mg/mL), with the injection volume being 10
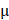
L/g of the mouse weight. PAM, Sorafenib and Sorafenib+PAM were subcutaneously injected into two spots of each tumor-carrying mouse with 100
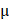
L/spot. Mice were sacrificed on the 18^th^ day starting from the date of recruitment, and the tumors were dissected (**Figure 6G**).

### Computational data and analytical tools

The whole transcriptome data used was retrieved from 14. Transcription factor prediction was performed using ConTra V3 36. Breast cancer relapse free survival was analyzed using Kaplan-Meier Plotter^73^. Gene expression profiles between breast cancer patients and normal individuals were visualized using GEPIA 74. E-MTAB-181 from ArrayExpress (http://www.ebi.ac.uk/arrayexpress) containing information on 56 breast cancer cell lines were used to access the differential gene expression profiles of genes under interest.

### Experimental data analysis

All experiments have at least three biological replicates. Normality was checked using the Shapiro-Wilks test. Two-tailed student's T-test was used to compare means between two groups. One-way analysis of variance (one-way ANOVA) followed by the Student-Newman-Keuls post hoc test was used for comparisons among three or more groups. Mean ± standard error of the mean (SEM) was used unless otherwise specified. The significance threshold used was p-value ≤ 0.5.

Data were analyzed and plotted using Graphpad prism 8.0.2.

## Supplementary Material

Supplementary figures and table.

## Abbreviations

ACSL1: Long-chain acyl-CoA synthetase 1
ACSL4: Long-chain acyl-CoA synthetase 4
AGPS: Alkylglycerone phosphate synthase
BQR: Brequinar
CAP: Cold atmospheric plasma
ChIP: Chromatin immunoprecipitation
CoQ: Ubiquinone
CoQH2: Ubiquinol
DAB: 3, 3'-diaminobenzidine
DAPI: 4', 6-diamidino-2-phenylindole
DEPC: Diethypyrocarbonate
DHODH: Dihydroorotate dehydrogenase
ER: Estrogen receptor
Fer: Ferrostatin-1
FSP1: Ferroptosis suppressor protein 1
GPX4: Glutathione peroxidase4
GSH: Glutathione
He: Helium
HER2: Human epithelial receptor 2
H_2_O_2_: Hydrogen peroxide
ICD: Immunogenic cell death
KEAP1: Kelchlike ECH associated protein1
Lip1: Liproxstatin-1
MS: Mass spectrometry
Nec1: Necrostatin-1
NO: Nitric oxide
NRF2: Nuclear factor erythroid2-related factor 2
O: Singleton oxygen
OH•: Hydroxyl radical
ONOOH: Nitrite in the form of proton
OONO^-^: Nitrite in the form of anion
O_3_: Ozone
O_2_^-^: Superoxide
PR: Progesterone receptor
PUFA: Poly unsaturated fatty acids
RFS: Relapse free survival
RONS: Reactive oxygenand nitrogen species
RSL3: RAS-selective lethal 3
SDS-PAGE: Sodium dodecyl sulfate-polyacrylamide gel electrophoresis
TNBC: Triple negative breast cancer
TRIM25: Tripartite motif containing 25
Zvad: Z-VAD-FMK
3ma: 3-methyladenine
