# Supplementary material for: Cold atmospheric plasma potentiates ferroptosis via EGFR(Y1068)-mediated dual axes on GPX4 among triple negative breast cancer cells
Source: Int J Biol Sci. 2025 Jan 1;21(2):874–92. doi: 10.7150/ijbs.105455 (PMC11705651; doi:10.7150/ijbs.105455)
Supplement: Supplementary file 1 — Supplementary figures and table. [file ijbsv21p0874s1.pdf]

Supplementary information

Supplementary Table 1. Mass spectrometry data showing proteins differentially interacted with EGFR in the wildtype and EGFR(Y1068F)-mutated SUM159PT cells.

Gene name	Protein molecular weight	Protein score	Sequence coverage (%)	Unique peptide number	Peptide number	Secondary spectra number	Abundance	Present in EGFR(Y1068F)
TOP1	90.7	517	20	15	15	16	356614725	0
H1-4	21.9	495	36	4	11	13	68862220.75	1
LIG3	112.8	462	11	7	7	8	28799782	0
H1-3	22.3	432	24	1	8	10	62433884	1
RPS17	15.5	364	50	6	6	9	82310909.78	1
MYH9	226.4	345	6	8	8	9	7434329.5	0
TUBB4A	49.6	326	29	1	9	11	1153474.875	1
STAU1	63.1	305	20	8	8	9	27133748.88	1
RPS15	17	235	55	6	6	9	35005539.13	1
EIF6	26.6	226	23	3	3	5	17267444.5	1
RPS12	14.5	221	38	3	3	4	5670833.813	1
XRCC6	69.8	217	16	6	6	6	11248297.38	0
XRCC5	82.7	206	5	3	3	4	4466033.625	0
HSPA1L	70.3	201	11	2	5	5	4139591.5	1
RPL26L1	17.2	185	23	1	5	5	3595233.25	0
RPL9	21.9	175	36	6	6	7	122687800	1
EXOSC10	100.8	169	5	3	3	3	28702043.81	1
RPL22	14.8	164	21	4	4	5	197511200.5	1
PWP1	55.8	160	18	6	6	6	19958237.5	1
HRNR	282.2	152	4	2	2	3	3804330.875	0
PRPF19	55.1	148	13	4	4	4	6663263.75	1
POLB	38.2	142	11	2	2	3	4593755.313	0
SRP14	14.6	138	46	4	4	5	17031955.75	1
SUB1	14.4	137	39	4	4	5	50000262.5	0
ANXA2	38.6	133	16	4	4	4	12164845.75	1
RPL22L1	14.6	132	20	2	2	3	4399767.875	1
RBM34	48.5	130	14	4	4	4	7480960.156	1
DHX37	129.5	130	5	4	4	4	8308590.281	1
ATXN2L	113.3	128	4	3	3	3	7162634.25	1
POLR1G	55	127	15	3	3	3	4919326.5	1
RPS25	13.7	126	28	5	5	5	312026133	1
RPS27A	18	125	30	4	4	4	34258093	1
PABPC4	70.7	124	7	1	3	4		1
REPIN1	63.5	123	5	2	2	2	3103558	1
G3BP2	54.1	123	6	2	2	3	9679366.125	1
MRPS26	24.2	121	14	2	2	2	2656672.938	1
DDX6	54.4	120	7	3	3	3	4171331.375	1
TMA16	23.8	113	11	2	2	3	10174998	1
HNRNPC	33.7	111	11	3	3	3	14998810.5	1
SPATS2	59.5	104	6	2	2	2	3007116.625	1
TRA2B	33.6	104	10	2	2	2	2819839.375	1
DNAJC21	62	102	3	1	1	1	1114452.375	1
DDX54	98.5	99	5	4	4	4	8170979.375	1
G3BP1	52.1	98	6	2	2	2	3700344.813	1
MVP	99.3	97	5	3	3	3	3000629.625	0
EXOSC3	29.6	94	8	2	2	2	11997644	1
DAP3	45.5	93	7	2	2	2	3581728.438	1
EXOSC4	26.4	93	11	2	2	2	8217267.25	1
EXOSC2	32.8	92	11	2	2	2	3322074.75	1
EPRS1	170.5	92	3	3	3	3	3514951.375	1
XRCC1	69.4	92	7	3	3	3	6957971.25	0
FLNA	280.6	92	2	3	3	3	2277262.688	0
ANXA2P2	38.6	91	6	2	2	2	4289092.875	0
PTCD3	78.5	90	3	2	2	2	4735789	1
KIF2A	79.9	89	6	4	4	4	11857530.38	1
COPA	138.3	88	2	2	2	2	3450072.625	1
LSM12	21.7	85	12	2	2	2	4110319.375	1
SLC25A6	32.8	85	12	3	3	3	14780377.38	1

LLPH	15.2	82	23	2	2	2	6323944	1
EIF2S3	51.1	81	10	3	3	3	7892742.125	1
FTSJ3	96.5	81	4	2	2	2	2206364.625	1
C11orf98	14.2	81	21	2	2	2	16693173	1
RPS29	6.7	80	32	2	2	2	31061416	1
TIA1	42.9	80	3	1	1	1	1518990.125	0
LGALS3BP	65.3	79	5	2	2	2	874432.2188	0
DIMT1	35.2	78	12	3	3	3	7782020.75	1
DNAJA3	52.5	78	7	2	2	3	4385313.75	1
MRPS22	41.3	78	7	2	2	2	2277619.125	1
EIF5B	138.7	78	2	2	2	2	1558839.563	1
PURA	34.9	76	10	2	2	2	3346326.813	1
EXOSC8	30	72	7	1	1	1	2633049.5	1
HNRNPR	70.9	71	4	2	2	2	7119598.813	1
MRPS11	20.6	68	16	2	2	2	2932612.688	1
PPP1CC	37	68	7	2	2	2	7136294.75	1
DHX57	155.5	68	2	2	2	2	2621199.75	1
STRAP	38.4	67	5	1	1	1	741223.5625	1
UBA52	14.7	67	13	1	1	1	4404763.5	0
STT3B	93.6	66	3	3	3	3	9771313.25	1
**TRIM25**	**70.9**	**66**	**2**	**1**	**1**	**1**	**1880321.875**	1
SKP1	18.6	66	7	1	1	1	1405405.875	1
MRPS14	15.1	64	12	1	1	1	1119417.75	1
VTN	54.3	63	3	1	1	1	3516938.5	1
CGAS	58.8	63	6	2	2	2	2196752.281	0
RRS1	41.2	61	5	1	1	1	356001.1563	1
RBM14	69.4	60	4	1	1	1	1778609.75	0
ADAR	136	59	2	2	2	2	415757.875	1
RRP8	50.7	59	4	1	1	1	627138.875	0
MAP7D1	92.8	57	4	2	2	2	1541875.625	1
SSBP1	17.2	57	10	1	1	1	2640765	0
NACA	23.4	56	7	1	1	1	2113308	1
ASPH	85.8	55	2	1	1	1	1101646.75	1
MOV10	113.6	55	4	3	3	3	6024166.125	1
RPS19BP1	15.4	54	10	1	1	1	4352406	1
EXOSC7	31.8	54	5	1	1	1	2359759	1
USP10	87.1	54	2	1	1	1	2092466.125	1
WDR5	36.6	54	4	1	1	1	867353.625	1
GPATCH4	50.4	53	8	2	2	2	4053408.75	1
SURF6	41.4	53	2	1	1	1	5917643.5	1
PHB	29.8	53	4	1	1	1	588069.9375	0
RUVBL2	51.1	52	6	2	2	2	2222922.125	1
DDX31	94	52	2	1	1	1	1007103.938	1
EXOSC9	48.9	51	3	1	1	1	6002844	1
LUZP1	120.2	50	3	2	2	2	1579977.375	1
MRPS5	48	50	4	1	1	1	722216.9375	1
RPL7L1	29.7	50	5	1	1	1	1033553.063	1
SRSF3	19.3	49	18	2	2	2	18412175.5	1
PURB	33.2	49	14	2	2	2	4094165.063	1
MRPS6	14.2	49	8	1	1	1	977594.125	1
STAU2	62.6	48	2	1	1	1	1765896	1
SRSF6	39.6	48	5	2	2	2	13277549	1
CCDC59	28.7	48	5	1	1	1	2050178.375	1
MRPS18B	29.4	48	6	1	1	1	2803518.75	1
FBL	33.8	46	9	2	2	3	15352501	1
RPS28	7.8	45	23	2	2	2	11631727.81	1
POLR1E	47.2	45	3	1	1	1	2788909.75	1
PRPF39	78.4	45	2	1	1	1	454593.75	0
SRPK1	74.3	44	5	2	2	2	1605945.875	1
C9	63.1	44	2	1	1	1	3262398	1
ABCF2	71.2	44	2	1	1	1	1158275	1
SRBD1	111.7	43	1	1	1	1	392474.5625	1
NOP53	54.4	43	4	1	1	1		1
SRP19	16.1	43	11	1	1	1	1130060	1
MRPS7	28.1	42	6	1	1	1	914610.125	1
RRP15	31.5	42	5	1	1	1		1
ARF4	20.5	42	6	1	1	1	2085451.75	1

SNRPF	9.7	41	15	1	1	1	1971385.5	1
YTHDC2	160.1	41	1	1	1	1	1833687.375	1
DUOX2	175.3	41	0	1	1	1	4621030.5	1
BOP1	83.6	41	5	2	2	2	2175823.875	1
GAPDH	36	41	7	1	1	1	1325120.5	1
NSUN2	86.4	41	1	1	1	1	1822705.375	1
CBLN4	21.8	41	3	1	1	1	72883056	0
DDX47	50.6	40	3	1	1	1	1397554.625	1
DDX56	61.6	40	3	1	1	1	250314.1406	1
GTPBP1	72.4	40	2	1	1	1	2151773	1
MGST1	17.6	40	10	1	1	1	3210816.75	1
RFC2	39.1	40	4	1	1	1	1328309.375	0
S100A6	10.2	39	9	1	1	1	11325717	1
YWHAQ	27.7	39	6	1	1	1		0
S100A9	13.2	39	11	1	1	1	986664.9375	0
PNKP	57	39	3	1	1	1	1369978.25	0
NT5C1B	68.8	38	1	1	1	1	16515932	1
DCD	11.3	38	13	1	1	1	3803610	0
ANKFY1	128.3	37	1	1	1	1	3246041	1
MRPS27	47.6	37	4	1	1	1	1703888	1
MYL6	16.9	37	9	1	1	1	3369671.75	0
AHNAK	628.7	37	1	1	1	1	695526.5	0
DARS1	57.1	36	2	1	1	1	1618755.5	1
NOL10	80.3	36	1	1	1	1	13594068	1
HSP90AB1	83.2	36	1	1	1	1		1
KARS1	68	36	2	1	1	1	420404.2813	1
TGFBI	74.6	36	2	1	1	1	767425.6875	0
MYH11	227.2	35	1	1	1	1	368444.25	1
MRPL41	15.4	35	11	1	1	1	249823.2344	1
IGLV2-11	12.6	35	6	1	1	1	10300549	0
LDHB	36.6	35	6	1	1	1	265015.8438	0
SRP68	70.7	34	2	1	1	1	549661.25	0
IGHG1	36.1	33	2	1	1	1	15873033	1
EIF2AK2	62.1	33	2	1	1	1	2140173	1
RPL37	11.1	32	6	1	1	1	68002960	1
NEPRO	64.5	32	2	1	1	1	1391610.125	1
MRPS34	25.6	31	12	1	1	1	978010.3125	1
BBX	105.1	31	1	1	1	1	986895.4375	1
SLC1A5	56.6	31	2	1	1	1	572575.6875	1
TGM2	77.3	31	1	1	1	1	490295.0625	0
CDC5L	92.2	30	1	1	1	1	1623419.375	1
KNOP1	51.6	30	3	1	1	1	3093323	1
MRPS2	33.2	29	4	1	1	1		1
IRS2	137.2	29	0	1	1	1	2425404.75	1
ELAVL1	36.1	29	4	1	1	1	1615446	1
SRPK2	77.5	29	2	1	1	1	1548335.625	1
TRIP4	66.1	29	3	1	1	1		1
RIOX1	71	29	2	1	1	1	1837230.75	1
PRRC2C	316.7	29	0	1	1	1	2899495.25	1
NOP58	59.5	29	2	1	1	1	1670212.5	1
LRRC59	34.9	28	3	1	1	1	4042896.25	1
ANKRD66	27.9	27	2	1	1	1	3404805	1
MPHOSPH6	19	27	7	1	1	1	2283442	1
VDAC2	31.5	27	3	1	1	1	1607393.375	1
PRDX1	22.1	27	5	1	1	1	6989649.5	1
SRP9	10.1	26	13	1	1	1	7328111.5	1
AP2M1	49.6	26	5	1	1	1	1057328.25	1
TUBA4B	27.5	26	5	1	1	1	5667833.5	1
MRPS28	20.8	26	6	1	1	1	1358786.875	1
SPOUT1	42	26	3	1	1	1	2382118.75	1
MEPCE	74.3	26	2	1	1	1	1279852.625	1
AP2B1	104.5	26	2	1	1	1	859802.6875	1
CSDE1	88.8	25	2	1	1	1	760543.0625	1
NIP7	20.4	25	8	1	1	1	2465024	1
RRP7A	32.3	25	5	1	1	1	2708624	1
SRSF9	25.5	24	5	1	1	1	3153678	1
ABLIM3	77.8	23	1	1	1	1	371245	1

SNRPB	24.6	22	3	1	1	1	5774359	1
RNGTT	68.5	21	2	1	1	1	8152892	1
BTBD17	52.4	21	1	1	1	1	27696956	1
ERH	12.3	20	6	1	1	1	2642483	1
CMAS	48.3	20	3	1	1	1	494441.9063	1

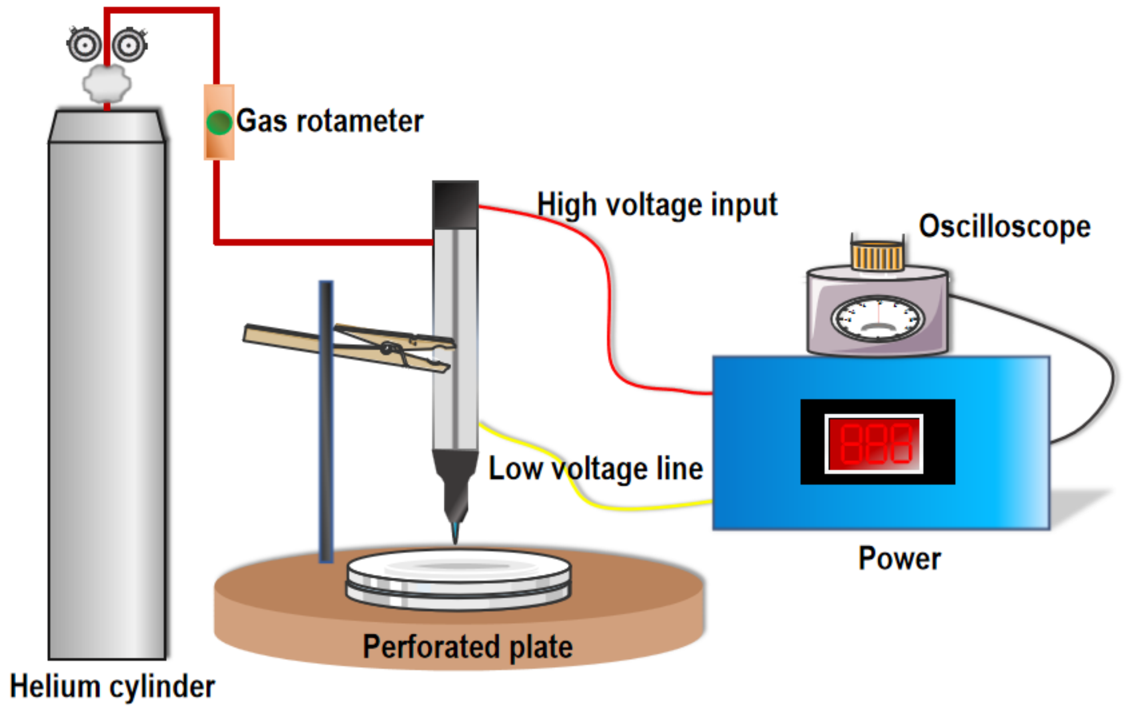

Supplementary Figure 1. Illustrative diagram of the home-made plasma source.

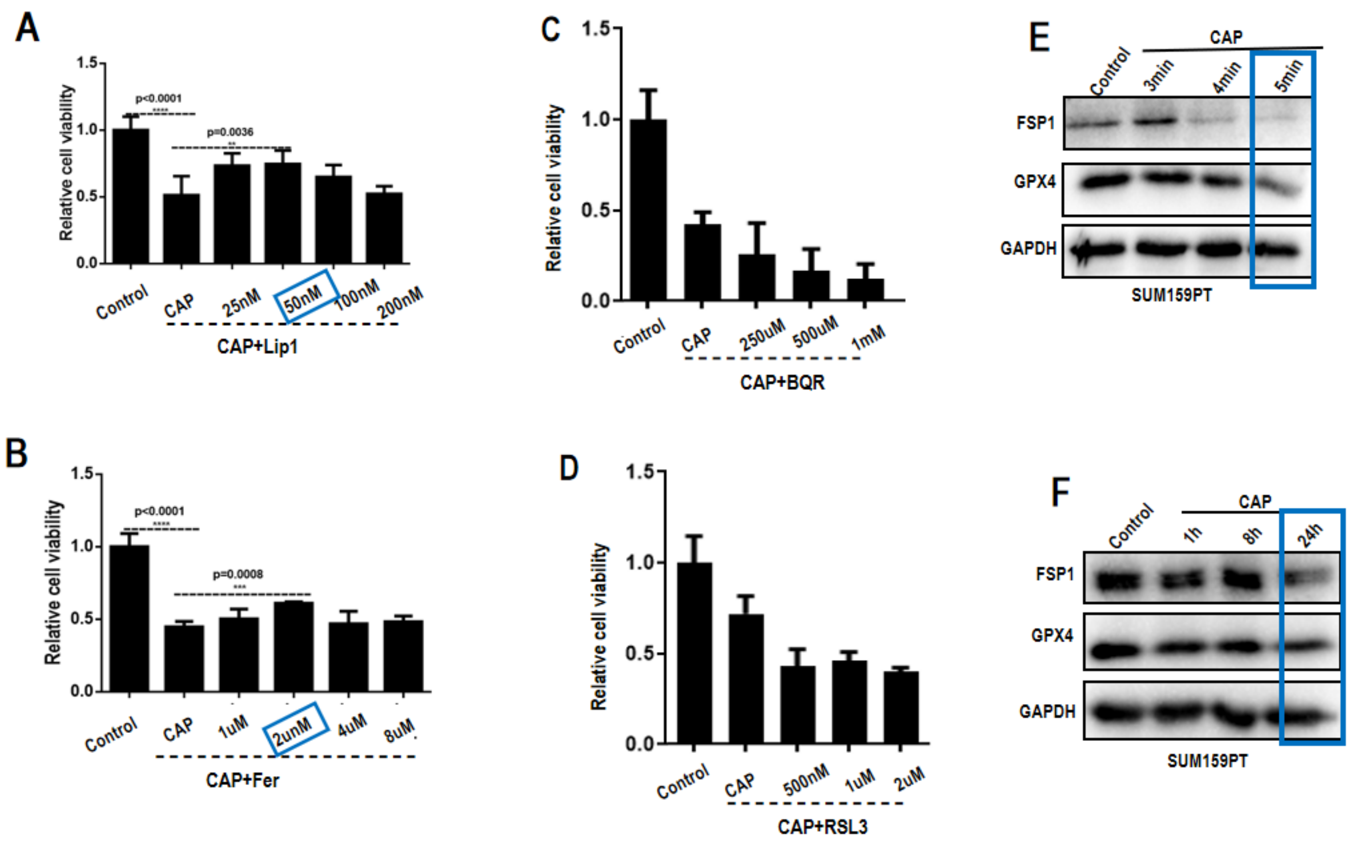

Supplementary Figure 2. Dosing effect of ferroptosis inhibitors and activators, CAP treatment and incubation durations on inducing ferroptosis in SUM159PT cells. Dosing effects of the ferroptosis inhibitors **(A)** liproxstatin-1 (Lip1), **(B)** ferrostatin-1 (Fer), and ferroptosis activators **(C)** brequinar (BQR) and **(D)** RAS-selective lethal 3 (RSL3) on the viability of SUM159PT cells in response to CAP treatment. Dosing effects of **(E)** CAP exposure duration and **(F)** incubation duration on the protein expression of canonical ferroptosis markers GPX4 and FSP1 in SUM159PT cells. SUM159PT cells were used. According to panels (A) and (B), 50 nM Lip1 and 2 μM Fer were selected; and according to (E) and (F), 4 min treatment duration and 24 h post-treatment incubation time were selected in this study.

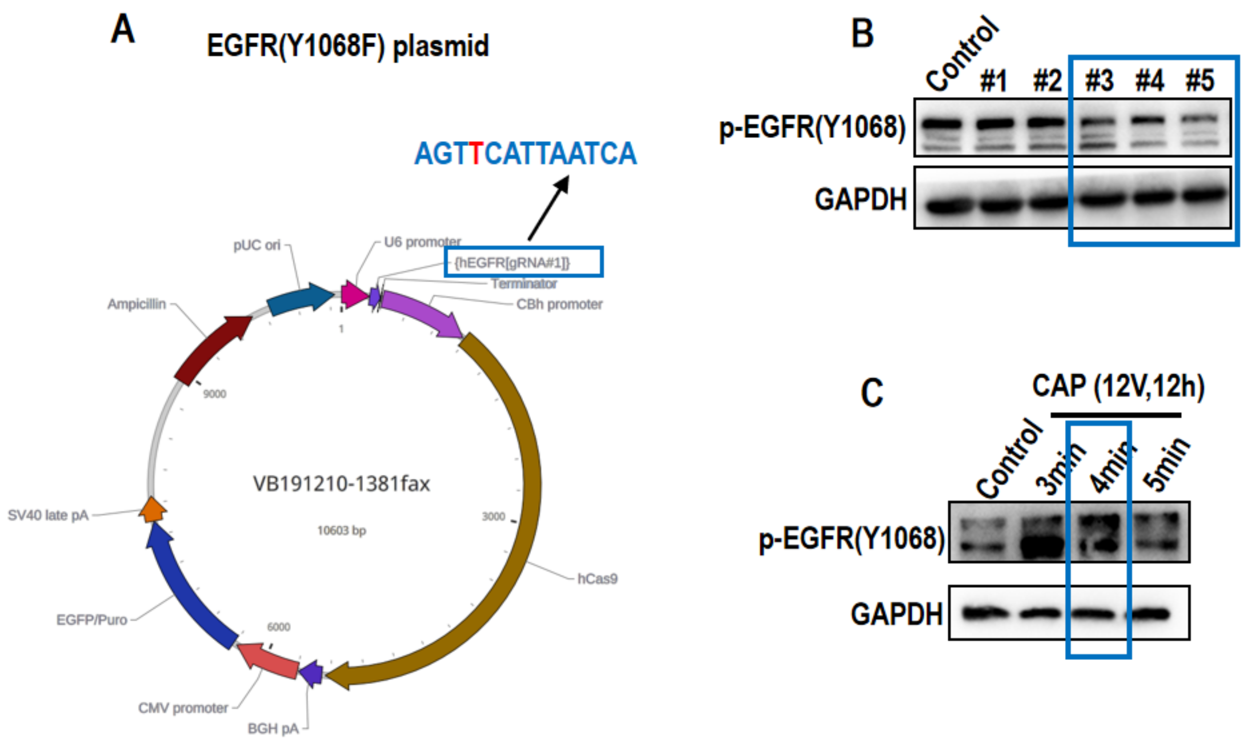

Supplementary Figure 3. Construction of the plasmids carrying EGFR(Y1068F) mutation and stable EGFR(Y1068F) mutant SUM159PT cells. (A) Plasmid design for generating EGFR(Y1068F) mutation. **(B)** Construction of stable SUM159PT cells carrying EGFR(Y1068F) mutation. Clones number 3, 4, 5 showed reduced EGFR(Y1068) phosphorylation that suggested successful construction of EGFR(Y1068F) mutants. **(C)** Dosing effects of CAP on activating EGFR(Y1068) in SUM159PT cells under varied CAP exposure durations, where 12 V and 12 h incubation duration were used as the parameter setting for CAP ejection.

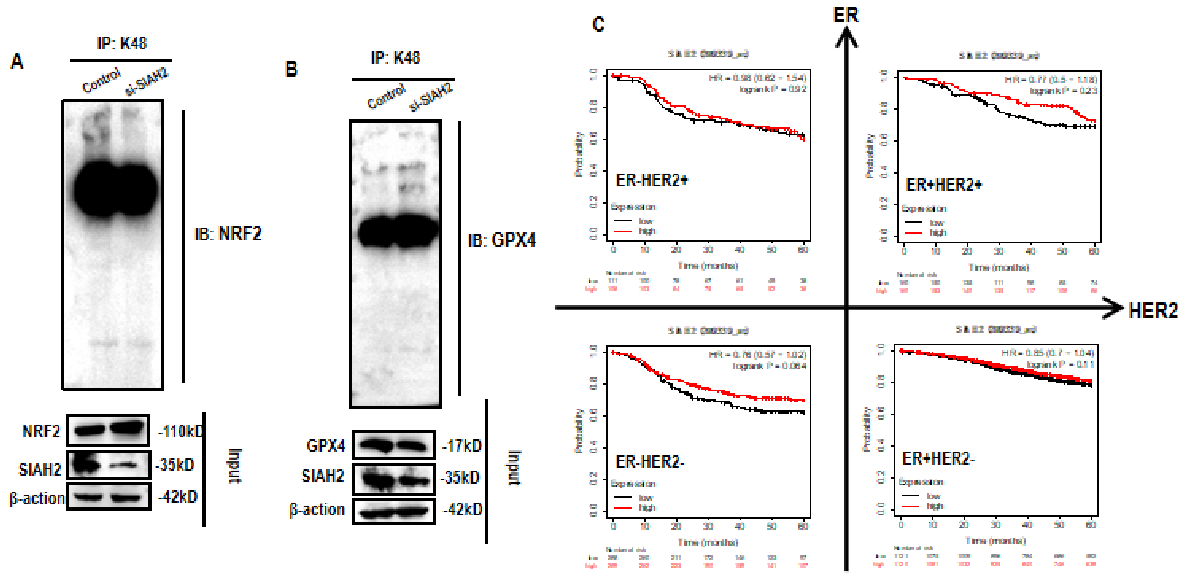

Supplementary Figure 4. Additional images for Figure 5. Full gel images showing immunoprecipitation results for **(A)** NRF2 K48 ubiquitination and **(B)** GPX4 K48 ubiquitination in SUM159PT cells transfected with *SIAH2* siRNAs. **(C)** Relapse free survival of different breast cancer subtypes as stratified by *SIAH2* gene expression using probe 209339 and analyzed using gene chip data stored in Kaplan-Meier Plotter[73].

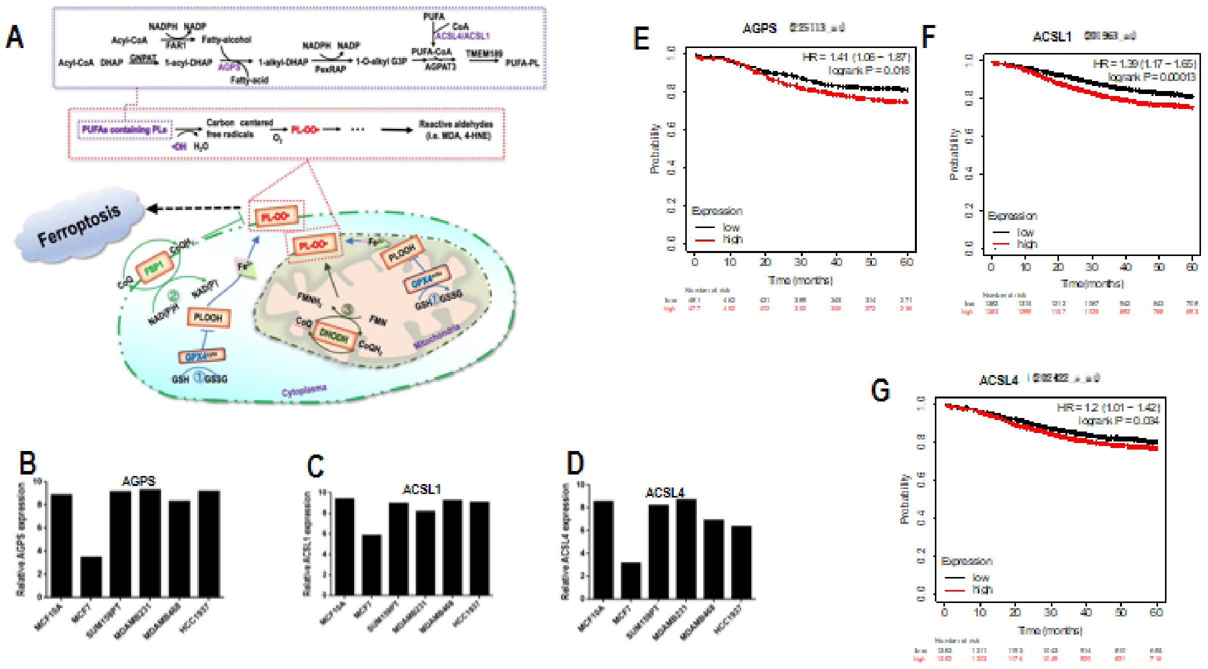

Supplementary Figure 5. Three pathways leading to ferroptosis and analysis of key enzymes controlling the synthesis of PUFAs containing PL. (A) Illustrative diagram of three ferroptosis pathways. The three pathways are characterized by GPX4 over-representation in cytoplasma and mitochondria inner membrane, FSP1 over-expression on cell membrane, and DHODH low expression in mitochondria inner membrane. The occurrence of ferroptosis requires both PL-OO· and PUFA, where the synthesis of PUFA is controlled by key enzymes such as AGPS, ACSL1, ACSL4. Gene expression of **(B)** AGPS, **(C)** ACSL1, and **(D)** ACSL4 in different breast cancer cell lines according to E-MTAB-181 data from ArrayExpress. Relapse free survival of breast cancer patients as stratified by **(B)** AGPS, **(C)** ACSL1, and **(D)** ACSL4 gene expression using Kaplan-Meier Plotter[73]. PUFA: polyunsaturated fatty acids; PL: phopholipids.
